# Evolution of risk preference is determined by reproduction dynamics, life history, and population size

**DOI:** 10.1038/s41598-017-06574-5

**Published:** 2017-08-24

**Authors:** Oren Kolodny, Caitlin Stern

**Affiliations:** 1 Department of Biology, Stanford University, Stanford, CA 94305 USA; 2 Santa Fe Institute, 1399 Hyde Park Road, Santa Fe, NM 87501 USA

## Abstract

Alternative behavioral strategies typically differ in their associated risks, meaning that a different variance in fitness-related outcomes characterizes each behavior. Understanding how selection acts on risk preference is crucial to interpreting and predicting behavior. Despite much research, most theoretical frameworks have been laid out as optimization problems from the individual’s perspective, and the influence of population dynamics has been underappreciated. We use agent-based simulations that implement competition between two simple behavioral strategies to illuminate effects of population dynamics on risk-taking. We explore the effects of inter-generational reproduction dynamics, population size, the number of decisions throughout an individual’s life, and simple alternate distributions of risk. We find that these factors, very often ignored in empirical and theoretical studies of behavior, can have significant and non-intuitive impacts on the selection of alternative behavioral strategies. Our results demonstrate that simple rules regarding predicted risk preference do not hold across the complete range of each of the factors we studied; we propose intuitive interpretations for the dynamics within each regime. We suggest that studies of behavioral strategies should explicitly take into account the species’ life history and the ecological context in which selection acted on the risk-related behavior of the organism of interest.

## Introduction

Over the course of their lifetimes, individuals are presented with decisions that entail a choice between a risk-prone strategy and a risk-averse strategy. For example, should an animal forage alone, running the risk of not finding food, but reducing competition for any food that it does find, or forage in a group, minimizing the probability of not feeding at all, at the cost of procuring only a small part of any prey item that is detected (e.g.^1, 2^)? Should Australian Martu women hunt for large but difficult-to-catch kangaroo, or ubiquitous but small goanna lizards^3^? Individuals vary in their propensity to select the riskier of the possible strategies at these day-to-day decision points, as well as in their risk-related choices at crucial junctures in their life history, such as mating^4^ and dispersal^5^, suggesting that risk-taking behavior can be subject to selection.

A wide range of causes that give rise to uncertainty are referred to as risks; here, we use *risk* to describe uncertainty in the outcome of a behavioral choice, as reflected in the fitness-determining character (*payoff*) that is influenced by that choice^6–10^. Two alternative behaviors can be considered *risk-averse* (or *low-variance*) and *risk-prone* (or *high-variance*) if the payoff from the first (in units of, for example, accumulated energy or contribution to the likelihood of successfully raising a fledgling) is always the same, whilst the payoff from the second behavior varies stochastically. The stochasticity in a *risk-prone* behavior can stem from sources such as food distribution; for example, foraging in an environment in which food is patchy is risky compared to foraging in an environment in which food is distributed uniformly. Similarly, difference in the risk associated with choice of a breeding site can stem from large variance between years in the suitability of some sites: one can imagine a site which is close to foraging grounds but highly exposed to climatic fluctuation, leading to very high reproductive success on some years but very low reproductive success on years in which one or two events of harsh weather occurred during the breeding season. Importantly, many alternative behavioral choices – even those that are not perceived as associated directly with risk-taking – have different variances in fitness-related outcomes, and so include an implicit risk factor (see Supplementary Section 1). *Bet-hedging* refers to alternative strategies that arise in response to selection on variance in payoffs, and its study overlaps significantly with that of risk sensitivity. We use throughout terms that are common in the risk-preference literature, and discuss our model in terms of bet hedging in Supplementary Section 6.

A variety of factors can influence the evolution of risk-taking, and have been dealt with extensively in models and in empirical studies (e.g. refs 11–23). A factor that has received relatively little attention for its role in shaping risk-taking strategies is the *selection regime*, or *reproduction dynamics*, that is, the algorithm by which an individual’s decisions or resource acquisitions are translated into offspring in the next generation. The importance of the reproduction dynamics is most obvious when considering both competition and evolutionary dynamics at the population level, whereas most frameworks that deal with risk taking have been built from the individual’s perspective and do not include population or reproductive dynamics (e.g. refs 6, 10, 24–26). Additionally, the influence of frameworks that treat population dynamics in the context of risk preference (see refs 27–32) has been limited in some scientific communities that study animal behavior.

Because the reproduction dynamics that a population experiences ultimately determine the fitness consequences of behavior, they can have important consequences for the evolution of risk-taking strategies^33^. Moreover, reproduction dynamics are expected to play a crucial role in the evolution of any behavior in which alternative strategies differ in the variance associated with their outcomes, i.e., that have an implicit risk factor. However, many studies of ecology and evolution of behaviour implicitly or explicitly assume certain reproduction dynamics without considering the extent to which they reflect the selection regime in the biological system that is explored. Here, we first study the two reproduction dynamics that are most commonly employed in mathematical models of animal behavior, proportional selection (e.g. in refs 34, 35) and truncation selection (e.g. refs 36–39), and then explore the effects of two alternative models that capture realistic aspects of selection in the wild, the first characterized by a convex or concave fitness function (as is assumed in many studies of optimal foraging^6, 7, 9, 40^), and the other characterized by a sigmoid fitness function (see, e.g., refs 41, 42).

In populations experiencing proportional selection, each individual’s contribution to the next generation is proportional to the value of the fitness-determining character (henceforth *accumulated payoff*, or just *payoff* ^30, 43^). In contrast, in populations experiencing truncation selection, only individuals whose accumulated payoff is above a certain threshold contribute offspring to the next generation^30, 44, 45^. Predicting the effect of reproduction dynamics on the fitness consequences of risk taking behavior is not always straightforward and may depend, for example, on the variance in payoffs of the risk-prone strategy as well as on details of the payoff distribution that are not captured by its variance; it may also depend on aspects of the organism’s ecology and life history such as the typical number of choices in life that are related to this behavior; and finally it may critically depend on population dynamics and population characteristics such as its size (see, e.g., refs 46).

Understanding of how reproduction dynamics influence the evolution of risk-taking is impeded by the rarity of studies comparing these dynamics within the same modeling framework and the paucity of studies that explore a range of parameters related to organisms’ life history and population dynamics. For example, most classic and recent models of the evolution of risk-related behavior employ proportional selection or a concave fitness function without comparing their effects to those of truncation selection^10, 15, 46^. Overall, proportional selection seems to be more commonly used than truncation selection in evolutionary models. The emphasis on proportional selection is appropriate if it is the most common in nature, but is this the case?

Truncation selection, or selection that is well approximated by truncation, may be more common than previously appreciated in natural populations. One broad category of species in which truncation selection occurs is those in which only individuals that surpass a specific body condition threshold are able to breed. Evidence for such reproductive thresholds has been found in organisms including female vipers (*Vipera aspis* ^47^), female albatrosses (*Diomedea exulans* ^48^), female sticklebacks, (*Gasterosteus aculeatus* ^49^) and male and female petrels (*Halobaena caerulea* ^50^). While some populations show evidence of the ability to adjust reproductive thresholds in the face of low resource levels^51^, reproductive thresholds nonetheless lead to restriction of the set of parents represented in the next generation. Truncation selection is also characteristic of species in which male-male competition and female choice lead to strongly skewed male reproductive success, in a non-proportional relation to those males’ measure of quality, such that only a small proportion of the males in a population produce any offspring at all. These dynamics occur in many species including rhesus macaques (*Macaca mulatta* ^52^), red deer (*Cervus elaphus* ^53^), and wild turkeys (*Meleagris gallopavo* ^54^).

Many species likely experience an intermediate form of selection that incorporates characteristics of both proportional and truncation selection. Birds, for example, are characterized by social monogamy, which occurs in more than 80% of bird species^55^. This reduces variance in male reproductive success relative to polygyny^56^ and – combined with females’ choice of partner based on perceived quality – leads to selection that is seemingly close to proportional. However, extra-pair paternity, which occurs in over 75% of socially monogamous bird species^55^, can increase variance in male lifetime reproductive success in a way that is disproportional to each male’s quality^57, 58^, shifting the system to a selection regime that may be nearer to truncation selection if the realized fitness function resembles a sigmoid or towards a fitness function that is entirely concave or convex. Thus, modelling approaches that use forms of selection intermediate between truncation and proportional selection or that gradually deviate from these simple schemes may most closely approximate the reproduction dynamics experienced by many species.

Beyond the implicit or explicit choice of reproduction dynamics, models of risk-taking often ignore reproduction altogether, and consider only the survival component of fitness^59–61^, even though the reproductive component in a whole-population context has a critical effect on evolutionary outcomes^25, 62, 63^. Additionally, some models focus on short-term rather than long-term effects of risk-taking, particularly in the context of foraging (e.g., refs 6, 10, 59, 60, 64). Although risk-taking in foraging, a task that is repeated many times in an organism’s lifetime, can have cumulative fitness consequences, the link between risk-taking at this scale and long-term reproductive fitness is often unclear^25^.

In this study, we use agent-based simulations of constant-sized populations to study the influence of reproduction dynamics on the evolution of behavior that has a risk component. Individuals in natural populations likely face a small number of decisions with large fitness consequences over the course of a lifetime, e.g., mating choice and breeding site selection, which could have a large effect on the number of offspring produced. These decisions are the focus of our model: we ask how reproduction dynamics influence lifetime fitness when risk affects an individual’s payoffs at key points in its life history (see also Supplementary Section 1). We discuss how our findings relate to behaviors that differ from these, i.e. behaviors in which the link between variance in payoffs and long-term fitness is more complex, such as foraging.

## Methods

In order to study the effects of population dynamics, and in particular of reproduction dynamics, on the extent to which different strategies of risk preference are favored by selection, we conducted agent-based simulations in constant-sized populations.

For transparency and tractability, we realized a highly simplified and general scenario of a haploid population with constant size (*N*), in which each agent’s behaviour is genetically encoded, and is either risk-prone (an individual that *plays the high-variance strategy*) or risk-averse (an individual that *plays the low-variance strategy*). Each time step in the simulation represents a generation, during which agents receive payoffs according to their behaviour in a number of events (*E*) throughout their lifetime. The number of events is a model parameter and is constant throughout each simulation run. These events can represent foraging bouts, nesting locations, or other fitness-related choices.

The payoff for risk-averse individuals is constant per event, whilst the payoff that a risk-prone individual receives in each event is determined by a random draw, and is either high or low. The probabilities of each occurrence are chosen such that the mean payoffs of the strategies are identical.

Generations are non-overlapping, and at the end of each time step the whole population is replaced by *N* new individuals^65^. Each individual in generation *t* + *1* is an offspring of an individual in generation *t*. Immigration and emigration are not considered. Parenthood is assigned probabilistically (a single individual may produce multiple offspring) and is related to the payoff that each individual had accumulated (see below).

In all the results reported below, runs were initiated with populations in which half the individuals were risk-prone and half were risk-averse. Unless noted otherwise, each simulation run was continued until the fixation of one strategy.

We implemented a number of reproductive schemes and compared their effects on selection for risk preference. It is important to note that, in a finite population, fitness is determined not only by the payoff scheme to the players of the different strategies and by the reproduction scheme, but also by the per-generation population-level state of affairs. In other words, the fitness to which a certain sum of payoffs would translate may depend on the particular distribution of payoffs that was stochastically realized in a certain generation in a simulation run. This highlights the importance of modeling the evolution of risk preference not only as an optimization problem studied from the point of view of an individual but in a way that explicitly considers population dynamics. A useful portrayal of this dependency is in game-theoretical terms: because the population size is kept constant, reproduction is a zero-sum game: the success of each player is necessarily on the expense of the others. Thus, for example, in an extremely small population, *N* = 2, in which proportional selection is realized (see below), in which one player received a payoff equal to 10 and the other a payoff equal to 5, the first player will be the parent of each of the individuals in the next generation with probability 10/(10 + 5). If, on the other hand, the second player received a payoff of 15 instead of 5, each individual in the next generation will be an offspring of player number 1 with probability 10/(10 + 15). Although player 1 received the same payoff in both cases, its fitness changed dramatically because of the fate of player 2.

### Proportional selection

In this reproduction scheme, the number of offspring that are probabilistically assigned to each individual is proportionally related to the sum of payoffs that the individual had accumulated during its lifetime. As noted earlier, this scheme is used as a default in many modeling frameworks.

### Truncation selection

In this reproduction scheme, all individuals are sorted according to the rank order of the sum of payoffs they had accumulated. Reproductive success is determined by a threshold: only the upper *T* % of the individuals in the population gives rise to offspring that constitute the next generation. Offspring are assigned at the same probability to all individuals that surpass the threshold, regardless of their sum of payoffs. As noted above, this reproduction scheme is frequently used in models because of the simplicity and efficiency of its implementation. It is also frequently used in artificial selection of animals and plants in agriculture^66, 67^. Although the efficiency of truncation selection on allele variation has been studied^44^, we do not know of a model of its effect on risk preference. See Supplementary Section 3 for a discussion of an alternative truncation selection scheme, in which the truncation threshold is set to a constant value of accumulated payoffs.

We also implemented two reproduction dynamics that are, in some respects, intermediates between proportional and truncation selection, and that capture realistic aspects of some species’ reproductive dynamics:

### Power-weighted selection

The number of offspring probabilistically assigned to each individual is proportional to the sum of its payoffs, raised to the power of *z*. For *z* > 1, this leads to a convex fitness function, and for *z* < 1, to a concave function (see Figures S5–S7 in Supplementary Section 4). The case of *z* = 1 represents proportional selection. As noted above, convex and concave fitness functions have been modelled extensively in theoretical frameworks. Our framework differs from these in its explicit consideration of population-level dynamics.

### Sigmoid-weighted selection

This reproduction scheme allows a gradual departure from the proportional selection scheme towards a fitness function that is more similar to the one created by truncation selection. The number of offspring probabilistically assigned to each individual is proportional to its sum of payoffs after the following transformation:

$$Weighted\,payoff=\frac{1}{(1+{e}^{(-\xi \cdot ({p}_{n}-\beta ))})},$$

where ξ represents the steepness of the sigmoid, *p*_*n*_ the individual’s sum of payoffs after normalization such that the highest-scoring individual has a sum of payoffs equal to 1, and *β* represents the sigmoid’s inflection point (see Figures S8,S9 in Supplementary Section 4).

In finite populations, any selection scheme determines individual reproductive success relative to others in the population. It is worth noting that some selection schemes do so based on the rank order of accumulated payoffs of the individuals (such as the truncation selection scheme that we have implemented) and others do so based directly on the accumulated payoff value (such as our scheme of proportional selection). Although beyond the scope of the current work, most schemes can be implemented with regard to either of the two alternatives, sometimes leading to different results with respect to risk-preference. Which of the two is more appropriate depends on the ecology of the species of interest. For example, in a polygynous species in which direct competition between males over females is a major determinant of reproductive success, it is likely that rank order will be more appropriate, whilst direct use of the accumulated payoff might be most appropriate for a species in which brood size is strongly correlated with parents’ physical condition.

To test whether simulation results are different from the expected null result, we applied one-tailed t-tests. Unless noted otherwise, all ‘statistically significant differences’ refer to Z-tests with p < 0.001. The terms ‘similar success’ and ‘no difference’ refer to statistically non-significant differences (p > 0.001).

## Results

### Proportional selection

#### One decision in a lifetime

We study the effect of population size on whether a risk-prone or risk-averse strategy is favored when individuals have one decision during their lifetimes, every risk-averse individual receives a payoff of 10 per event, and every risk-prone individual receives a payoff of 5 or of 15 per event at an equal probability. We find that, for all simulated population sizes (*N* = 10, 50, 100, 250, 500, or 1000), the risk-averse strategy is significantly more likely to fix in the population, with probability of fixation ranging from 53% to 56% of simulations (significantly greater than 50% at p < 0.001, with non-significant differences in fixation probability between population sizes, at p > 0.05, Figure S1). The advantage a risk-averse strategy has under this scenario, and its independence of population size, are qualitatively supported by a simple derivation (Supplementary Section 1). These results are in line with the predictions of Gillespie’s model^27^, who suggested that selection in favor of strategies that minimize variance in offspring numbers is a general evolutionary principle. This stems from the finiteness of the population, which determines that the frequency of a strategy is a concave function of its payoff; Jensen’s inequality dictates, in such cases, that the strategy with lesser variance will have an advantage^28, 68^. It also aligns with the general finding that selection in settings with inter-generational variance in the number of offspring dictates the maximization of the geometric mean^32, 68^. Hintze *et al*. found somewhat divergent results from ours, in which population size affects risk preference, due to small differences in the studied dynamics^28, 46^. Notably, the advantage of a risk-averse strategy in our simulations is significant but small, and in many simulations the risk-prone strategy is the one that fixes in the population (see also ref. 27).

This finding, the frequent fixation of the suboptimal strategy, runs counter to the common expectation that observed risk-preference will usually align with the predicted optimal behavior (see discussion in ref. 22). It suggests that caution is warranted in deriving predictions regarding the expected risk preference in natural populations: the evolutionary dynamics of any single population or species – analogous to a single simulation run – are likely to have been quite different from the mean trajectory that is calculated over a large number of simulations. See Supplementary Section 6 for a discussion of our model in the context of other frameworks.

#### Multiple events in a lifetime

When individuals experience multiple strategy-choice events in their lifetime, the difference between the risk-prone and risk-averse strategies diminishes as the number of decisions grows, from a fixation probability of the risk-averse strategy of 54% in a simulation with a single event per lifetime (significantly different from 50% at p < 0.001) to a fixation probability that is not significantly different from 50% (at alpha = 0.001; *N* = 100; Figure S2). This result can be explained in terms of variance: under proportional selection, for a wide range of scenarios, a high-variance strategy is expected to be selected against^28^. This variance is most pronounced for a single lifetime event, and as the number of events per lifetime increases, the variance of the normalized sum of lifetime payoffs diminishes as a result of the law of large numbers (see discussion in Supplementary Section 2; see also ref. 46).

It is important to note that the variance in the risk-prone payoff in these simulations is quite high compared to the mean payoff, and accordingly the risk-averse strategy’s selective advantage is well within what is typically considered the regime of “strong selection”^27^. Interestingly, the selective advantage decreases to a non-significant level fairly quickly as the number of events increases, and is indistinguishable from no selective advantage even when the number of events per lifetime is as small as 10 (p > 0.5; *N* = 100, number of simulations = 5000).

Counter to some portrayals (discussed in, e.g., ref. 22), this finding suggests that risk-averse behaviors do not have a selective advantage in the context of frequently-recurring activities such as foraging bouts, if the activities’ payoffs are accumulated towards a long-term goal such as reproduction or migration (risk sensitivity may, for example, arise as a result of selection for energy-gain aimed at short-term survival, which imposes a qualitatively different selection regime. See discussion below).

#### The effect of different payoff distributions on the dynamics of proportional selection

To tease apart the effect of the risk-prone payoff’s distribution around the mean from the effect of the payoff’s variance, we conducted simulations in which the distribution of risk-prone payoffs was, as before, bimodal and discrete (individuals receiving a high payoff at a certain probability and a low payoff at a certain probability), but asymmetric, i.e., we conducted sets of simulations in a range of scenarios which differed from one another in the probability with which the risk-prone players received the high payoff. The probabilities we explored were P_high_payoff_ = {0.1, 0.2, 0.3, 0.4, 0.5, 0.6, 0.7, 0.8}. In each set of simulations, we adjusted the size of the payoffs such that the expected mean payoff would always be 10 (equal to the payoff of the risk-averse strategy), and the variance in payoffs would be identical to the variance in the baseline scenario described in the sections above. Individuals in all simulations experienced a single strategy-choice event in life.

We find that, in these simulations, the results do not differ from those found for the basic scenario described earlier: in all simulations (*N* = 100, 5000 simulations per parameter set) the probability of the risk-averse strategy’s fixation does not significantly differ from 0.55 and significantly differs from 0.5 (Figure S3). However, when variance is not maintained, this pattern changes (Figure S4), and as the variance in the risk-prone players’ payoff increases, the risk-averse strategy has a greater advantage. These results align with the theoretical predictions^28^.

Intuitively, and as opposed to the common focus of risk-sensitivity frameworks on the individual’s perspective, these results can be best understood from the perspective of population-level dynamics: in proportional selection, the number of players of a certain strategy in the next generation depends linearly on the current-generation sum of payoffs of players of that strategy; thus, if the probability of the two risk-prone payoffs varies between scenarios, but the variance does not change, the expected distribution of the *sum* of risk-prone players’ payoffs does not change, and all scenarios lead to the same result: the difference between the scenarios is in the distribution of payoffs among the risk-prone players, which has no effect on the population-level outcome. When the variance in payoffs is not kept constant, however, the expected distribution of the sum of risk-prone players’ payoffs is different for each scenario, and so the extent to which the risk-averse strategy is preferred varies.

### Truncation selection

#### Truncation threshold of 50%

**Figure 1:**
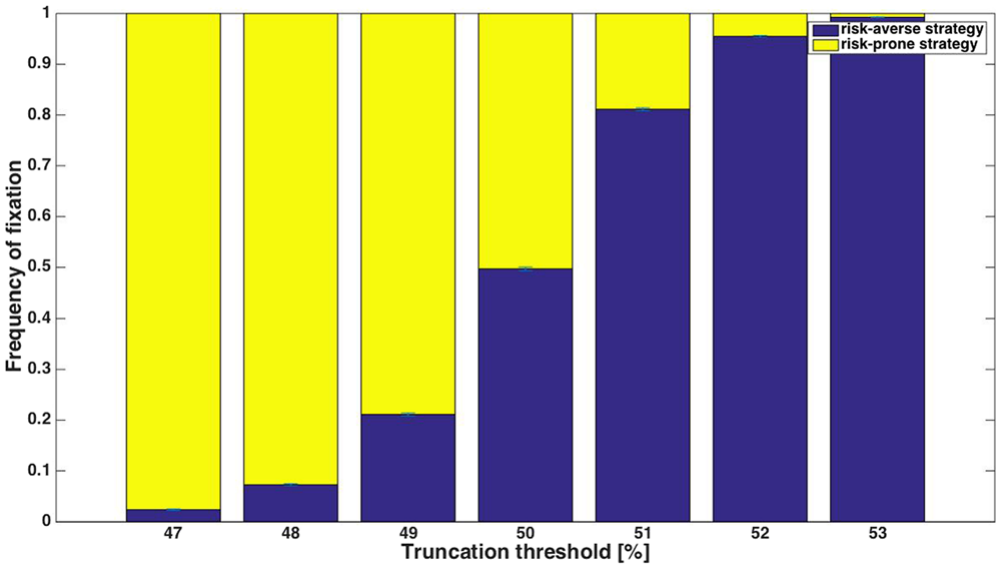
Truncation selection in which only the best-performing individuals reproduce, in a payoff scheme of 5-10-15 and a single choice during an individual’s lifetime. The X-axis depicts the truncation threshold; thus, for example, the leftmost bar represents the scenario in which the 47% individuals in the population that received the highest payoff are allowed to reproduce.

**Figure 2:**
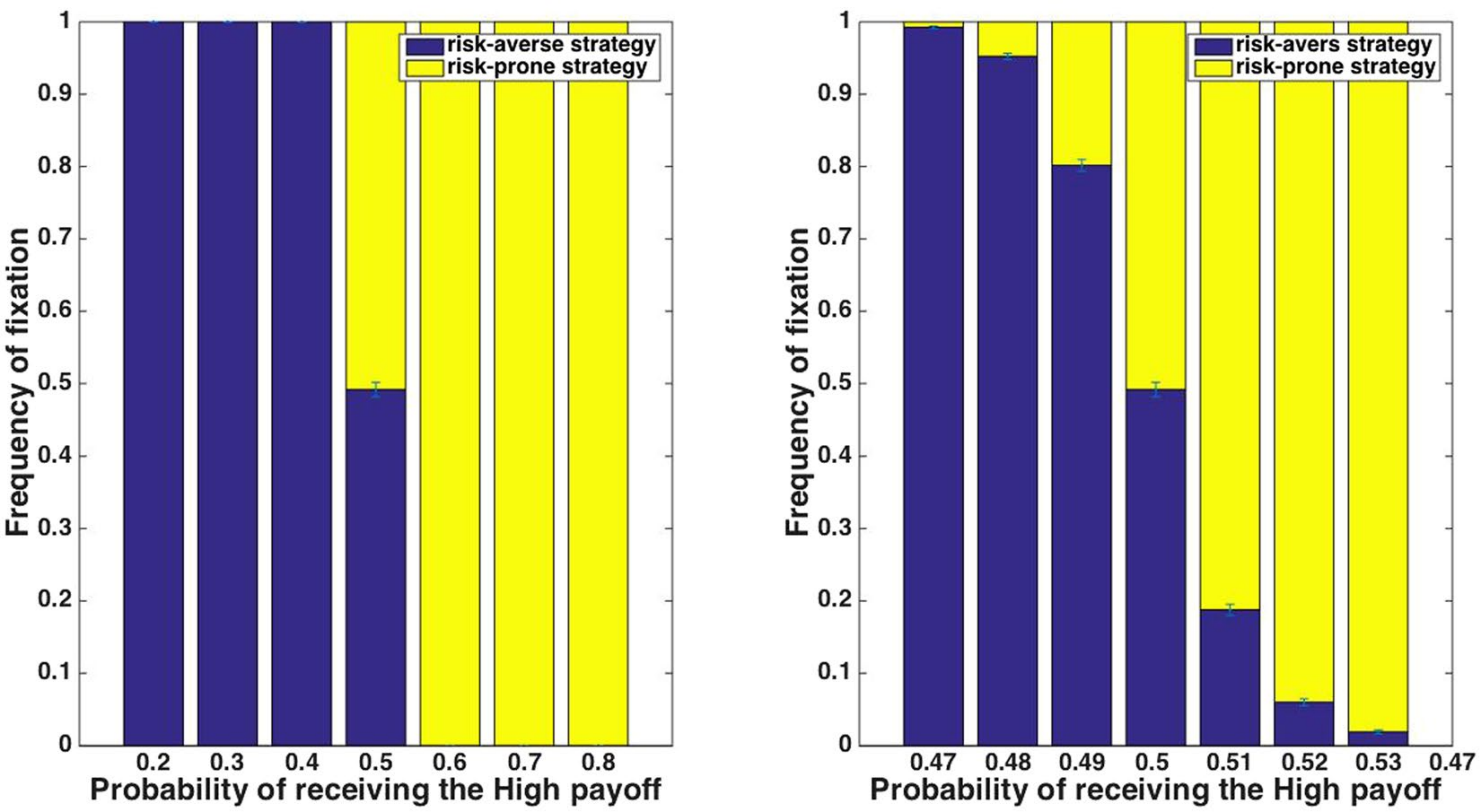
Truncation selection with various payoff schemes whose means and variances are the same, using a reproduction threshold of the 50% of the population with the highest payoffs. When the risk-prone strategy’s payoffs are distributed asymmetrically around the payoff mean, there is a higher probability of receiving one of the two payoffs. This leads to a strong advantage to the risk-prone strategy, dependent on the probability of the risk-prone players’ receiving each of the possible payoffs. Panel (a) shows payoff schemes that differ in the probability of risk-prone players’ receiving the high payoff; panel (b) shows results for payoff schemes in which the probability of receiving the high payoff is near 0.5.

When the truncation threshold is 50% (i.e., every individual that gains a payoff above average can reproduce), a single choice-event occurs per generation, and the payoff scheme is 5-10-15 (players of the risk-prone strategy receive a payoff of 5 or of 15 at equal probabilities, and risk-averse players receive a payoff of 10) neither of the two strategies has an advantage (Fig. 1). This result might seem to suggest that truncation selection at a 50% threshold is – as opposed to proportional selection – insensitive to risk. This turns out to be incorrect: for almost any other payoff scheme, even those in which there is an equal mean payoff to the two strategies, truncation selection at 50% leads to a *strong* bias in favor of one of the two strategies (Fig. 2): whether the risk-averse or risk-prone strategy prevails depends upon the frequency with which the two alternatives in the risky scenario occur. If the risk-prone individual is more likely to receive the high payoff than the low payoff, the risk-prone strategy has an advantage over the risk-averse strategy, and fixes in almost all simulation runs. The opposite scenario, in which the low payoff occurs with high probability, leads to the fixation of the risk-averse strategy in nearly all runs.

Although the high sensitivity to potentially very small differences in payoff probabilities may seem non-intuitive at first, these results are easily explained: in this scenario, an individual’s fitness is fully determined by its location above or below the reproductive threshold, with no influence of the *extent* to which it is above or below it. Thus, the evolutionary success of the risk-prone strategy depends merely on whether individuals that use it have a greater than 50% chance of getting a higher payoff than that of risk-averse individuals. Since the mean payoff is the quantity that is held constant in these simulations, any case in which the high and low payoffs are not symmetric around the mean leads to a trivial advantage or disadvantage to the risk-prone strategy, which accordingly fixes or goes extinct.

These results also hold for simulations with more than a single choice-event per lifetime, although they are gradually attenuated as the number of events increases: as seen with proportional selection, as the number of events per generation increases, the sum of payoffs that determines fitness converges towards the mean payoff, and thus alternative strategies become gradually more similar in their success.

Importantly, the variance in the payoff of the risk-prone strategy in all of the payoff schemes used above (Fig. 2) is the same. This demonstrates that – as opposed to proportional selection, whose outcome was sensitive to variance but not to other aspects of the payoff distribution – truncation selection is highly sensitive to the distribution of payoffs, but to components other than variance per se, a possibility that is rarely considered in studies of risk sensitivity or bet hedging.

#### Truncation thresholds other than 50%

For the payoff scheme of 5-10-15 (and all others in which the risk-prone strategy’s payoffs are symmetric around the mean), any truncation threshold at a value higher than 50% selects for risk-averse strategies (e.g., if the best-performing 75% of the population reproduce uniformly, all risk-averse players will always reproduce), while a threshold lower than 50% selects for risk-prone strategies. If, for example, only the best-performing 10% of individuals reproduce, those 10% will nearly always be composed solely of risk-prone players, because approximately half of the risk-prone players are expected to receive the high payoff.

This is not the case for other payoff schemes. In fact, it turns out that the effects of truncation selection *cannot* be easily summarized by a simple rule-of-thumb such as “truncation at a threshold above 50% leads to risk preference”: the evolutionary outcome of dynamics with truncation selection depends on the combined effect of the threshold value and details of the distribution of payoffs to players of the risk-prone strategy. For any truncation threshold, including 50%, and even when the strategies have the same mean payoff, there are payoff distributions that lead to the risk-averse strategy being favored, and other payoff distributions that lead to the risk-prone strategy being more advantageous. Thus, for example, for a threshold of 20%, most payoff schemes that maintain a mean of 10 and variance of 25 lead to fixation of the risk-prone strategy in the vast majority of simulation runs, but this is not the case for the payoff scheme of 8.333-10-25, in which the risk-averse strategy fixes in nearly all simulation runs.

### Realistic dynamics: deviations from proportional and truncation selection

One might argue that, although widely applied in studies, neither of the two schemes explored so far reliably mirrors realistic reproduction for most species in nature. We explore two additional families of selection schemes that come closer, we believe, to representing real-life reproduction in a range of species.

#### Power-weighted selection (convex/concave fitness function)

Under this selection scheme, each individual’s probability of reproduction is proportional to its accumulated payoffs, to the power of *z*, a simulation parameter. The value of z determines whether the fitness would be convex (*z* > 1) or concave (*z* < 1).

**Figure 3:**
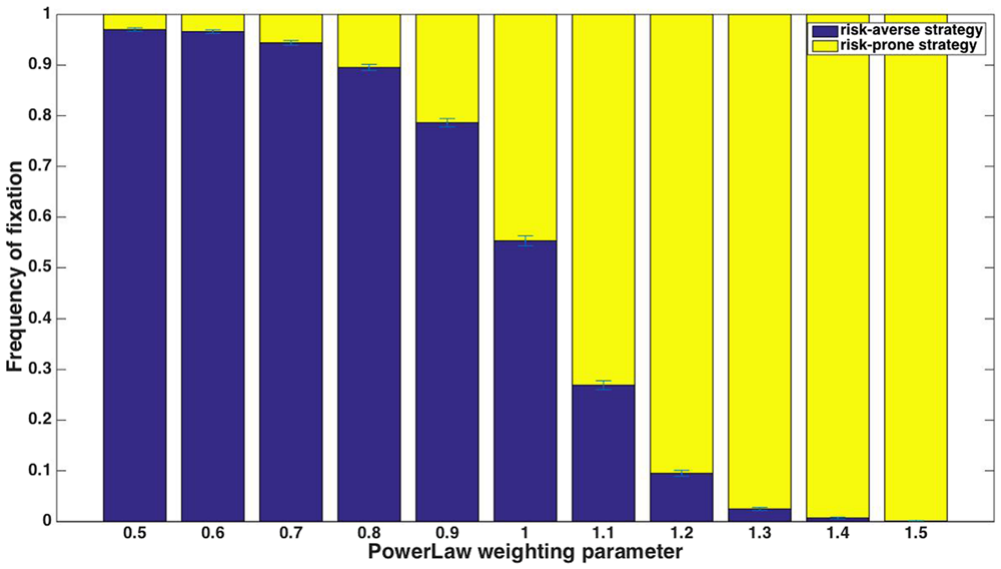
For a power-weighted selection scheme, with a payoff scheme of 5-10-15 and a single decision during an individual’s lifetime, the risk-averse strategy is favored when the fitness function is concave (*z* < 1), and the risk-prone strategy is favored when the fitness function is convex (see deviation from this rule near *z* = 1, below).

Our simulations under this scheme give rise to results that are in line with standard theory, i.e. a convex fitness function leads to an advantage to the risk-prone strategy while a concave function favors the risk-averse strategy (e.g. refs 10, 40; Fig. 3; but see also an exploration of a slight deviation from this regularity below). This is explained by Jensen’s inequality (see, e.g., ref. 68).

This qualitative result is robust to many details of the system: not only is it true for *z* values below and above the threshold of *z* = 1, we find that it also holds for different distributions of risk in the risk-prone alternative, i.e., it is true not only for the special case in which the high and low payoffs are symmetric around the risk-averse payoff. The same qualitative result is also found for any population size: in this scenario, a large population does not attenuate the effect of the payoffs’ variance on the relative success of the two strategies.

**Figure 4:**
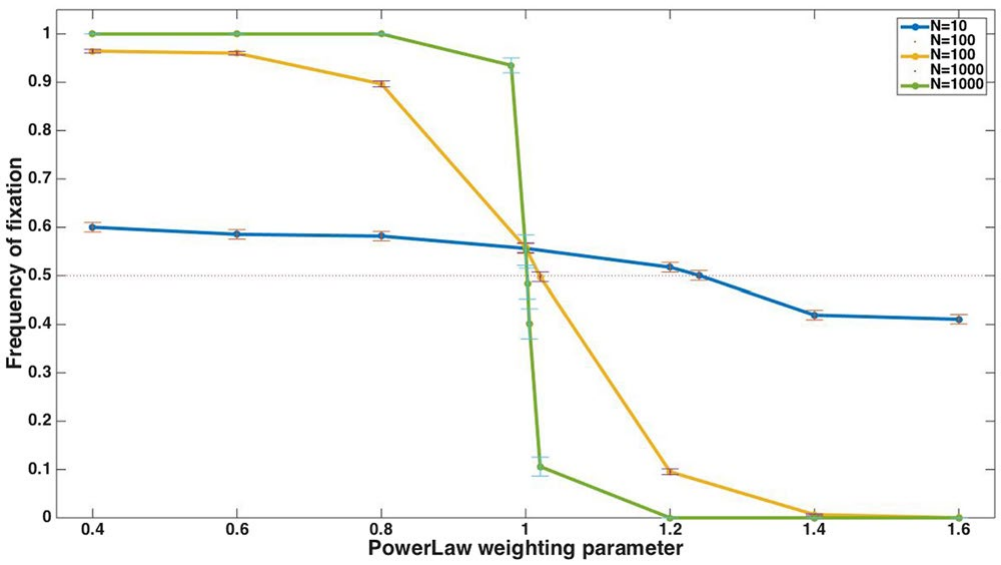
The frequency of fixation of the risk-averse strategy in a power-weighted selection scheme for a range of parameters, which give rise to convex or concave fitness functions. Qualitatively, concave functions lead to an advantage to the risk-averse strategy and convex fitness functions lead to an advantage to the risk-prone strategy, but the details of the system are important: the extent to which one strategy is preferred over the other strongly depends on the population size.

This robustness of the qualitative result may, on the other hand, be misleading: only the qualitative result is insensitive to the details of the system, while the quantitative results, i.e., the extent to which one strategy is preferred over the other, depends critically on these details, which have not been considered in previous studies. For example, we find that for a population size of *N* = 1000, a symmetric distribution of the risk-prone payoffs around the mean, and *z* = 1.26, the risk-prone strategy fixes in all simulation runs (*n* = 10,000 simulation runs). With a population of *N* = 100, the risk-prone strategy fixes in 95.5% of runs, and for a population of *N* = 10, the risk-prone strategy fixes in only 52.5% of runs. Population size influences which strategy fixes because the efficiency of selection depends on the population size^65^: in large populations, random drift is weak relative to selection, while in small populations fixation occurs within a small number of generations, and stochastic changes in strategies’ frequencies have a large effect. Figure 4 shows the probability of fixation of a risk-prone strategy for three population sizes over a range of parameter values that determine the extent to which the fitness function is convex or concave.

A value of *z* = 1 equates this selection scheme with proportional selection, explored earlier. We have shown that this scheme, in finite populations, leads to a small advantage of a risk-averse strategy over a risk-prone one. This slight advantage may be offset by a slight convexity of the fitness function, whose magnitude depends on the population size; thus, for each population size, the value of *z* at which the risk-prone and risk-averse strategies are equally likely to fix is slightly different. This can be seen in Fig. 4.

As opposed to predictions that are derived for infinite populations and to some general derivations outside of a population context, when the risk in the risk-prone payoff is extremely high, meaning that one of the payoff options is very rare, exceptions to the general rule occur: a risk-prone strategy may fix fairly frequently even in a scenario with a concave fitness function, and a risk-averse strategy may fix fairly frequently even in a scenario with a convex fitness function. This is particularly true in small populations, in which the effects of stochasticity are large. This occurs because the rare payoff may stochastically not be realized by a representative fraction of the risk-prone population, leading to atypical dynamics. Thus, for example, in a population of N = 50, a payoff scheme of 5-10-500, and *z* = 1.5, although the risk-prone strategy is expected to have a strong advantage, it fixes only in 27% of simulation runs.

#### Sigmoid-weighted selection

A realistic fitness function for many organisms is likely to be well-approximated by a sigmoid. At the limit, this scenario approaches a truncation selection scheme, which is a perfect step function. Notably, in our implementation (see Methods), the truncation selection scheme differs quantitatively from the sigmoid fitness scheme in that truncation is strictly based on relative ranking among the individuals in the population, whilst an individual’s fitness under the sigmoid-weighted selection scheme does not depend on its rank in the population. Importantly, an individual’s reproductive success depends on that of the rest of the individuals, both via the constraint that population size remains constant and via the dependency of the sigmoid’s form on the best-performing individual in the population: following the classic definition, fitness is normalized in every generation such that the individual with the highest normalized payoff in the population, *p*_*n*_, will have a fitness of 1.

**Figure 5:**
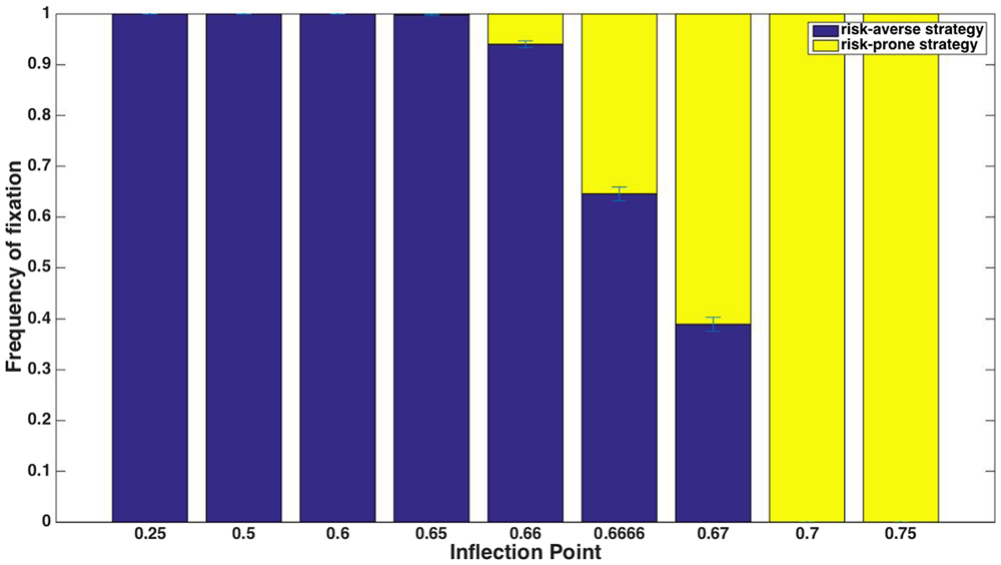
Under a sigmoid-weighted selection scheme, the inflection point of the sigmoid has a strong influence on which strategy is preferred. For the payoff scheme of 5-10-15 with a single decision during an individual’s lifetime, if the sigmoid’s inflection is above 2/3, the risk-prone strategy is favored, and if it is below, the risk-averse strategy is favored.

In a similar fashion to that seen in truncation selection, for a given payoff scheme, the determinant that governs which strategy is favored is whether players of each of the strategies are more likely to receive a payoff that is above or below the sigmoid’s inflection point. For the 5-10-15 payoff scheme and a constant steepness parameter of the sigmoid function, an inflection point at 2/3 is the tipping point: in simulations in which the fitness function has a higher point of inflection the risk-prone strategy is preferred, and the risk-averse strategy is preferred when the inflection is at *p*_*n*_ *< *0.66666 (Fig. 5). The extent to which the risk-averse or risk-prone strategy is favored is influenced by the steepness parameter and the population size.

**Figure 6:**
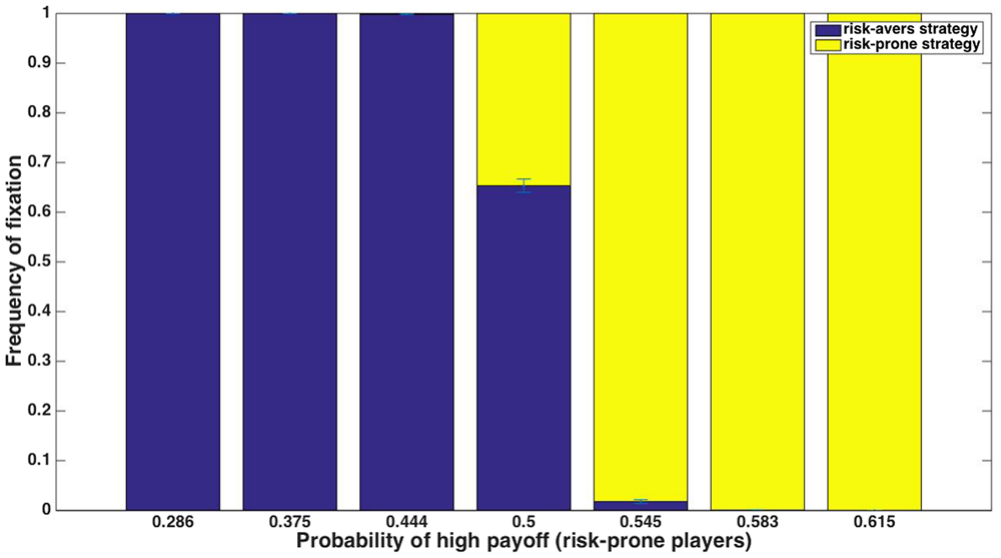
In a sigmoid-weighted selection scheme in which the inflection point is aligned with the risk-averse players’ payoff, the probabilities of the risk-averse players’ payoffs determine which strategy would be favored: if the payoff to the right of the inflection point is more frequent, the risk-prone strategy is favored, and otherwise it is selected against.

For interpretability, we explored a range of payoff schemes in which the higher payoff of the risk-prone strategy is constant at 15, and thus the typical inflection point of the sigmoid function (steepness, ξ = 10, inflection point, β = 0.66666) did not vary between the scenarios (Fig. 6). In line with the intuition provided above, in these simulations we find that, since the risk-averse players’ fitness is typically at the sigmoid’s inflection point, which strategy is preferred is determined by whether the high risk-prone payoff is more or less frequent than the low payoff. In simulations with different parameters from these, such as when the high risk-prone payoff is not fixed at 15, or when the inflection point is different from 2/3, a useful intuition is that whether the risk-averse payoff is typically higher or lower than the value of the inflection point usually determines whether the risk-averse strategy will be favored or selected against, respectively.

Exceptions to these generalizations are to be found, such as when the risk-prone payoffs are extremely asymmetric around the value of the risk-averse payoff, leading to a situation in which either of the two payoffs is realized only very rarely. This affects the outcome both by directly adding stochasticity, which may have a large influence when population sizes are small, and in leading to reshaping of the sigmoid function, which is normalized according to the highest-performing individual’s payoff, significantly altering the fitness value of each payoff between generations.

Most importantly, this analysis demonstrates that it is difficult to reach general conclusions about whether a certain sigmoid fitness function is likely to favor a risk-prone strategy over a risk-averse one or vice versa: the details of the payoff distribution, as well as population size and the ensuing probability that the high risk-prone payoff will be realized, determine which strategy will be favored. For any given sigmoid fitness function, payoff distributions can be found that would favor risk-taking or risk aversion.

## Discussion

Our simulation model reveals that reproduction dynamics (the inter-generational sampling process), life history, and the distribution of payoffs for risk-prone individuals (*players*) all influence the extent to which a risk-prone strategy is favored over a risk-averse strategy or vice versa, suggesting that the consideration of population-level dynamics in studies of behavior is crucial. Below, we highlight the major results and draw insights that are important for both empiricists and theoreticians studying the evolution of behavioral strategies that differ in their expected variance, i.e. that incorporate – explicitly or implicitly – an element of risk.

Our results align with the findings of existing frameworks with regard to proportional selection and to concave and convex fitness functions^10, 28, 68^, yet highlight underappreciated aspects of these settings. To our knowledge, truncation selection and a sigmoid fitness function have not been studied in the context of risk preference. Our findings in these scenarios differ qualitatively from those of proportional selection, highlighting the importance of their consideration (see Supplementary Section 5). Our simple model allows straightforward explanation of the findings, which are not surprising from a theoretical standpoint but nonetheless have important implications that seem to be underappreciated. Our studies’ relation to existing literature is further discussed in Supplementary Section 6.

Our results also demonstrate the importance of the number of decision events in an individual’s lifetime, and the difference in relative variance in overall payoff between the strategies, as well as the particular distribution of the possible payoffs, in determining whether a risk-averse strategy is favored over a risk-prone one. Under proportional selection, we find that a risk-averse strategy is favored over a risk-prone strategy when individuals only make one decision per lifetime, but the difference in success between the strategies diminishes as the number of decisions that individuals make during their lifetime grows. This is due to the reduction in the relative variance in overall payoff between the strategies, driven by the law of large numbers: the more decisions a risk-prone player makes, the more closely will the variance in its overall normalized payoff approach that of a risk-averse player (which, in the studied payoff schemes, is zero).

This result has implications for the decision contexts in which risk-taking behavior is expected to have significant fitness consequences. Because foraging typically consists of multiple bouts, representing multiple decisions, it is likely that in most species, over intermediate and long time periods, risk-prone and risk-averse foraging strategies show only a minor difference in overall variance. It is thus likely that risk preferences in foraging contexts should be explained by, for example, differences between individuals’ past experiences (e.g. refs 22, 33, 59, 69–72), cognitive biases^22, 73, 74^, or selection on short-term survival^22, 40, 59, 75^, which is strongly dependent on the species’ ecology, and not by direct selection on long-term reproductive output or a similar long-term measure of fitness as is the focus of our simulations (see also^29, 62, 76^ and Supplementary Sections 1 and 7).

Strong selection on risk preference is likely to arise when individuals differ in risk-taking with respect to a decision that occurs very infrequently during a lifetime but that may strongly influence its long-term fitness, such as the selection of a breeding site or selection of a mate. Identifying which decisions in an individual’s lifetime are infrequent, how the number of these decisions differs between species, and the extent of risk that is associated with alternative choices are empirical lines of investigation that will reveal the opportunity for alternative risk-taking strategies to arise (e.g. ref. 76). Although the idea that individuals may take different risks in different life history stages is well-established (e.g. ref. 77), the idea that the number of major decisions per lifetime influences the evolution of risk-taking behavior has been underappreciated and requires further exploration. Expansion of models that include only one decision per lifetime to include multiple decisions would aid in this effort.

Modeling evolutionary dynamics requires a choice of reproduction dynamics and thus brings about challenges for both empirical and theoretical studies. Empiricists should use caution when applying predictions from models to their systems whenever alternative strategies differ in variance, because of the sensitivity of evolutionary dynamics to the assumed fitness function. In particular, the widespread use of proportional selection in many models, which is reasonable because of its simplicity, may be inappropriate for a variety of taxa^52–54^. Rather, many real populations likely experience a non-linear effect of each individual’s payoff on its fitness, with some degree of truncation selection, with many individuals in the population failing to produce offspring altogether; this is especially likely in populations that exhibit a pronounced skew in reproductive success, including socially monogamous species with extra-pair paternity (e.g. refs 57, 58, 78). In theoretical studies, using a proportional selection regime as a default instead of explicitly taking into account the details of the reproduction dynamics in the modeled biological system may be problematic: variance in the payoffs of different strategies will influence which strategy is predicted to succeed. If variance is not the main factor of interest, its influence on the simulation outcome may obscure the results.

Under truncation selection, we find that whether a risk-prone or risk-averse strategy is favored is determined by both the truncation threshold (the proportion of the population that is allowed to reproduce) and the probability with which a risk-prone player acquires its low versus high payoff. All else being equal, the likelihood that a risk-prone strategy is favored increases as the proportion of the population that is allowed to reproduce decreases. However, the likelihood that a risk-prone strategy is favored decreases as the probability that it receives a low payoff for any single decision increases, even when the mean payoffs for risk-prone and risk-averse players are equal. Thus, even when only a small proportion of the population is allowed to reproduce, a risk-averse strategy could be favored if risk-prone players are unlikely to gain the higher of their two possible payoffs. This result indicates that the relationship between truncation threshold and selection for risk-taking behavior is not as straightforward as intuition suggests. Dekel & Scotchmer^79^ similarly found that, in finite populations, truncation selection does not automatically lead to increased success for risk-prone strategies; Winterhalder *et al*.^80^ also emphasized the importance of understanding the payoff distribution when studying the evolution of risk-taking.

As opposed to proportional selection, which was rather robust to the details regarding the source of variance, truncation selection is sensitive to these details: different distributions of risk, even when variance is held constant, lead to differences in the favoured strategy. This is because, in truncation selection, the impact of individuals’ payoffs is not additive: payoffs are first translated to the reproductive success of each player individually, and the strategy’s success is the sum of those individuals’ reproduction. In proportional selection, the distribution of payoffs among individual players that use each strategy does not matter; the sum of payoffs of the players of each strategy can be translated into that strategy’s success. This difference from proportional selection is true for the other schemes that are similar to truncation selection in that a non-linear transformation on the payoffs takes place in order to translate payoff into reproductive success. This reasoning suggests that only proportional selection is robust to the detailed characteristics of the variance in the payoff scheme. This feature of proportional selection is attractive from a modelling perspective, but since proportional selection might not be a realistic approximation for a broad range of systems, its use may lead to qualitatively incorrect findings.

The reproduction dynamics in many real populations may best be approximated as intermediate between truncation and proportional selection. Our tests of intermediate reproduction dynamics reveal that, when the relationship between payoff and fitness is non-linear, the success of the risk-prone strategy is strongly influenced by the probability of gaining each of the risk-prone payoffs, as was the case for truncation selection. For example, when capturing the relationship between payoff and fitness as a sigmoid function, in which the truncation threshold is less dependent on individual rank than under true truncation selection, we find a complex relationship between details of the reproduction dynamics and the benefits of risk-prone behavior, and an important role for the probability distribution of payoffs in determining the success of risk-prone players. This finding emphasizes the importance of understanding the ecology of focal species, in order to accurately estimate the probability distribution of payoffs and selection dynamics in the environment.

A sigmoid-weighted fitness function may be realistic for many organisms, and the insights derived from our implementation of this function deserve special notice. As opposed to other forms of selection, such as proportional selection, a sigmoid fitness scheme has a number of variables that may have a large effect on the effective selection regime that it defines. For example, whether the function is normalized such that the best-performing individual will have a fitness of 1, or whether some other benchmark for normalization is chosen, or whether the function is not normalized and is defined relative to absolute payoff values, may have a large impact on which risk preference will be favored for a given payoff scheme. We chose one possible implementation of a sigmoid function in order to explore its qualitative effect, but careful notice is warranted to the details of the chosen function in any study that applies it, taking into account the biological determinants that may govern the sigmoid’s form. For example, if the sigmoidal shape is a result of nutrient limitation in the environment, it may be preferable to set the sigmoid’s shape based on absolute payoff values, regardless of generation-to-generation differences in payoffs in the population. If a major factor in determining an individual’s fitness is direct competition, such as whether a male succeeds in establishing a territory, the function should perhaps be reshaped based on the realized distribution of accumulated payoffs per generation.

Our most striking finding is that, in the most realistic selection scenario, in which the fitness of individuals in the population is distributed along a sigmoid function, no simple generalization about risk-preference can be made. For any combination of selection parameters (i.e. the form of the sigmoid function), risk preference is dependent on the specific payoff distribution that is considered, and vice versa: for any distribution of payoffs, whether risk-taking is favored by selection or selected against depends on the details of the fitness function. This suggests that an explicit analysis and discussion of the various parameters of the system, including the characteristics of the population and the selection acting on it, is instrumental in any attempt to model the evolution of alternative behavioral traits that differ in their associated risks.

Our findings also underline the crucial role that the population context plays in determining a strategy’s success. We have explicitly shown that population size may affect outcomes under some selection schemes; more generally, the variance in an individual’s reproductive success and the relative fitness conferred by a certain strategy are dependent on the fitnesses (and hence strategies) of all other individuals in the population^8^. Assuming constant fitness through an allele’s sweep, drift, or loss from the population means that a model may yield predictions different from those of a model that takes the population context into account. Model assumptions that lead to individual fitnesses that are independent of the state of the population, as in some models of risk-taking in foraging (e.g. refs 59, 60), will likely yield incomplete results, because the probability of success of one strategy compared to the others depends on population-level processes. Our approach of modelling a finite population explicitly, using simulations, allows inclusion of the population context, and our analysis highlights the impact that the relative success of individuals can have on the long-term evolution of risk-taking behaviour under various schemes.

A factor that our model does not take into account, but that has been predicted and shown to significantly affect risk preference, is the individual’s state (e.g. refs 10, 32, 81): whether it is preferable to choose a risk-prone strategy may depend on the individual’s previously-accumulated payoffs as well as other parameters. For example, the probability distribution of possible payoffs of each behavioural choice is likely to be different depending on an individual’s body size, experience, or sex. Combining these with the factors considered in our model is an important next step. We predict that the importance of the factors whose role our study highlights, and in particular the strong dependency of risk-preference selection on the reproductive dynamics, will be heightened in models that consider state-dependence in the payoff distribution and that allow it to affect individual’s behavioural choices dynamically. This is because compared to proportional selection, the non-proportional selection schemes that we study over-weight the influence of the realized payoffs of all individuals in the population on each individual’s success. Accordingly, risk preference that considers individual state, particularly relative to others, is likely to be highly advantageous and play an even more prominent role than it does in proportional selection.

Another exciting avenue for future exploration is the evolution of the selection dynamics themselves: a species’ reproductive dynamics are an emergent property of its ecology, life history, and behaviour, all of which may change over time. One can imagine, for example, a species which initially is characterized by proportional selection, but whose dynamics gradually change towards increasing reproductive skew as a result of the interaction between the reproductive dynamics and the heritable traits for which they select.

Given that a selection regime intermediate between truncation and proportional selection is likely most realistic, does our analysis of intermediate regimes lead to any general conclusions about the circumstances that favor risk-taking? A rule-of-thumb indicated by our results is that the closer the selection regime is to truncation selection with a high threshold (only a few individuals are expected to parent a disproportional fraction of the next generation), the more likely it is that risk-taking will be favored. When the selection regime is instead more similar to proportional selection or truncation selection with a low threshold to reproduction, it is more likely that a risk-averse strategy will be favored. However, our main insight is that the favored strategy depends on the details of the alternative strategies, particularly the location of the payoff of the risk-averse players along the realized fitness curve, and the distribution of the payoffs to risk-prone players. Understanding the ecology and natural history of species studied is required in order to make predictions about the conditions under which risk-taking is expected to evolve.

## Electronic supplementary material

Supplementary Information
