# Supplementary material for: Evolution of risk preference is determined by reproduction dynamics, life history, and population size
Source: Sci Rep. 2017 Aug 24;7:9364. doi: 10.1038/s41598-017-06574-5 (PMC5571215; doi:10.1038/s41598-017-06574-5)
Supplement: Supplementary file 1 — Supplementary Information [file 41598_2017_6574_MOESM1_ESM.pdf]

Supplementary information for "Evolution of risk preference is determined by reproduction dynamics, life history, and population size", authored by

Kolodny, O. & Stern, C.

Supplementary contents

Supplementary 1: On the pervasiveness, time-scale, and risk-magnitude of risk-related behaviors

Supplementary 2: Additional results of proportional selection

Supplementary 3: A note on truncation selection

Supplementary 4: Realized fitness curves

Supplementary 5: the effect of population finiteness

Supplementary 6: A few comments that may help navigate related theory

Supplementary 7: The relation between risk-related behaviors that occur in key points of life and risk-related behaviors that recur frequently

Supplementary references

Supplementary section 1: On the pervasiveness, time-scale, and risk-magnitude of risk-related behaviors

It is frequently underappreciated that an element of risk, a difference in the expected variance in payoffs that is associated with alternative behavioral choices, is found in almost every choice that an organism faces. The magnitude of each decision's effect and the frequency and time scale on which they play out are dispersed across a vast range, from the difference in the expected variance in food acquired throughout a day to the difference in the variance of the expected lifetime reproductive success. For clarity, we outline a few very specific examples of such behavioral choices, in addition to the general examples found in the main text:

1. **Choice of breeding site**: Imagine a generalist species of amphibian that breeds in water. In many areas, breeding sites are available both in the form of seasonal ponds and of year-round water bodies. Frequently, in year-round water bodies tadpoles are exposed to high levels of predation by fish, leading to low but predictable amphibian reproductive success. Choosing to breed in seasonal ponds, on the other hand, may be a risk-prone strategy, as there are years in which the ponds dry up before tadpoles manage to metamorphose while on other years the ponds hold water for a longer period, allowing metamorphosis of the vast majority of tadpoles. The reproductive success of individuals that choose one strategy compared to the other may in some years differ by orders of magnitude.
2. **Mating strategy:** Alternative mating strategies exist in many taxa (reviewed in, e.g., [1]) that differ significantly in their variance: one can imagine a system in which males may invest much time and effort in mate-guarding a single female, which is likely to lead to successful mating and high paternity in the brood of offspring, or alternatively attempt to mate with multiple females and not mate-guard any of them, a strategy which can lead to a wide range of outcomes in terms of reproductive success, from very low to very high, depending on multiple factors.

3. **Dispersal:** In many species, individuals may remain near their natal site or disperse, sometimes to great distances. Without going into the details of the vast range of possible strategies and dynamics involved, one can imagine a simple scenario in which the choice is dichotomous and occurs at a certain point in life, typically upon reaching sexual maturity: remain in the natal locality and experience relatively predictable conditions (which would lead to low variance in expected lifetime reproductive success), or disperse, a choice which may lead to highly unpredictable outcomes (associated with high variance in the expected reproductive success).
4. **Social behavior:** In many social species, individuals copy others' behavior to learn or to capitalize on the successful choices made by others. This is done by, for example, scrounging for the food others detect or following the path that others chose. Whether to use this strategy at all, as opposed to alternatives such as self-exploration, and how to implement it if it is chosen, such as which individuals to copy, is almost always associated with difference in expected variance between the various alternatives.

In each of these behavioral contexts, the payoff influences lifetime reproductive success somewhat differently; some examples lend themselves more readily to interpretation in the terms of our model than others. To capture the precise link between the behavioral context of interest and each behavior's effect on fitness might require a specific model that describes the organism's ecology. We designed our model to be simple and general such that a very broad range of examples like these can be interpreted in the model's terms. For a broad range of behaviors (e.g. examples 1-2 above), the link of variance in payoff to our model's payoffs can be interpreted thus: each behavior's outcomes affects the number of offspring that an individual manages to produce and that reach some intermediate phase in life (metamorphosis in amphibians, fledging in birds, and so on). In their next phase of life, these offspring enter the pool of juveniles or of young adults, and only some will reach maturity and reproduce. This latter phase is captured by the inter-generational reproductive schemes that our study explores. Other behaviors, such as the dispersal example above, may be similarly interpreted in terms of our model

(in this case: if dispersal distance is small enough such that the offspring can be thought of as competing for the same limiting resources that define the inter-generational reproductive dynamics), but may also take place on a spatial or temporal scale that is much broader (as in long-distance dispersal, e.g. [2–7]) or much shorter (as in minute-by-minute foraging decisions) than the simple dynamics described by our model. We suggest that these are likely to have qualitative commonalities with our findings, but the details of each behavior must be considered for this to be determined. The Discussion and supplementary section 7 below discuss further the link between our findings and those of foraging contexts or other behaviors that play out on a very short temporal scale.

Supplementary section 2: Additional results of proportional selection

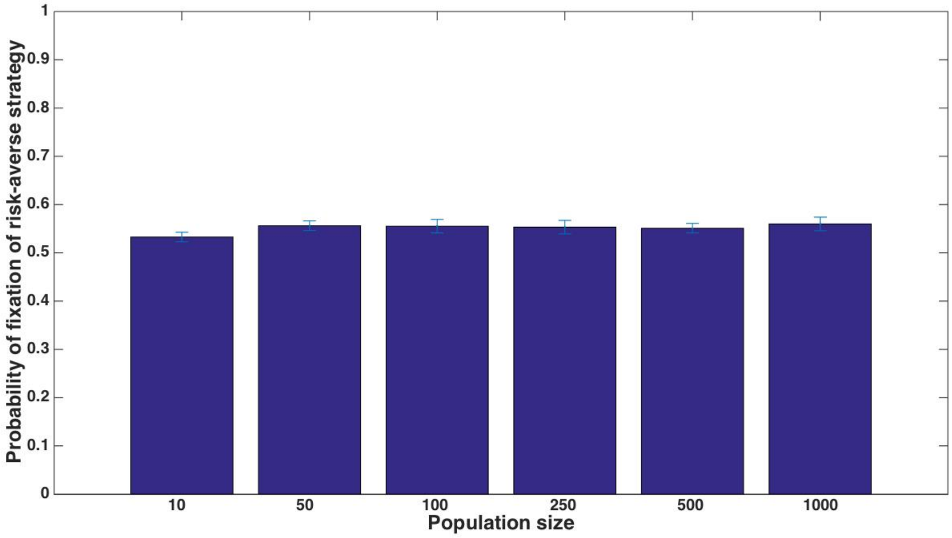

Figure S1: The probability of fixation of the safe strategy under proportional selection, for a payoff scheme of 5-10-15 (risky players receive a payoff of 5 or 15, and a mean of 10; safe players always receive a payoff of 10), and a single decision in an individual's lifetime. For all finite population sizes, the safe strategy is slightly preferred, and fixes in approximately 55% of simulation runs.

The finding that, under proportional selection, a safe strategy has an advantage over a risky one with an identical mean payoff, is in line with general analytical results that address the effect of variance on fitness in finite populations[8]. An analytical derivation that analyzes the particular scenario that we explore is beyond the scope of the current study, but a simple analytic analysis of the first step in each simulation can provide some intuition for our finding.

All simulations start with an identical number of individuals that play each strategy. Let us denote this number as K (and thus the population size is $2*K$). Under proportional selection, the expected fraction of players of each strategy in the next generation will be the expected sum of payoffs of players of that strategy in the first generation divided by the overall payoff that all players got. The sum of payoffs of risky players is the sum of payoffs of the x individuals that received the high payoff,

and the $K - x$ individuals that received the low payoff. For an infinite K, the expected ratio between this sum and the overall payoff in this generation would be equal to half, but in finite populations this is not the case. To calculate it exactly, we can use the fact that the distribution of x values follows a binomial distribution; for a given value of K we can calculate the probability with which x takes on each of its possible values. For each of these we can calculate the expected fraction of the overall payoffs that were received by risky players, and calculate the sum of these, weighted by their various probabilities. This provides us with the expected fraction of risky players at the end of the first generation. This calculation shows that for any K, the safe players' expected sum of payoffs is greater than half of the received payoffs in that generation. This advantage, which is 0.53333 for K=1, decreases as K increases.

Although describing only the first step of a simulation of the studied scenario, this derivation provides an intuition regarding the source of the advantage that a safe strategy has over a risky one in a finite population. It also raises the question: why does the advantage of the safe strategy, as measured in the probability of fixation in our simulations, not decrease as the population size increases?

This can be intuitively explained by a consideration of the overall selection dynamics: under a fairly broad range of conditions, efficiency of selection increases with population size [9]. Thus, although the fundamental advantage of the safe strategy stems from the deviation of a binomial draw from a normal distribution and thus decreases as population size increases, this decrease is balanced by an increase of the efficiency of selection for the small advantage of the safe strategy. An alternative intuition regarding this process is that the time until fixation of one strategy when the two strategies have a similar fitness coefficient is governed by drift, and thus scales with population size; the longer fixation time in small populations provides more opportunities for even a slight advantage of one strategy over the other to be realized. We do not derive the exact mode by which the two effects scale with population size, and note that their rates may not be perfectly aligned to completely cancel one another out, but their opposing directions may explain why an effect of increased population size on risk preference is greatly attenuated in the parameter space that we explore (see also discussion in [8]).

These intuitions also provide an explanation to our finding that, when population size is kept constant, the probability of the safe strategy's fixation decreases towards 0.5 as the number of decisions throughout an individual's lifetime increases (Figure S2). The increase in number of decisions reduces the deviation of the mean sum of payoffs of the risky players from the payoff that the safe players receive, but in this case, there is no change in the population size that would balance this reduction in the safe strategy's advantage.

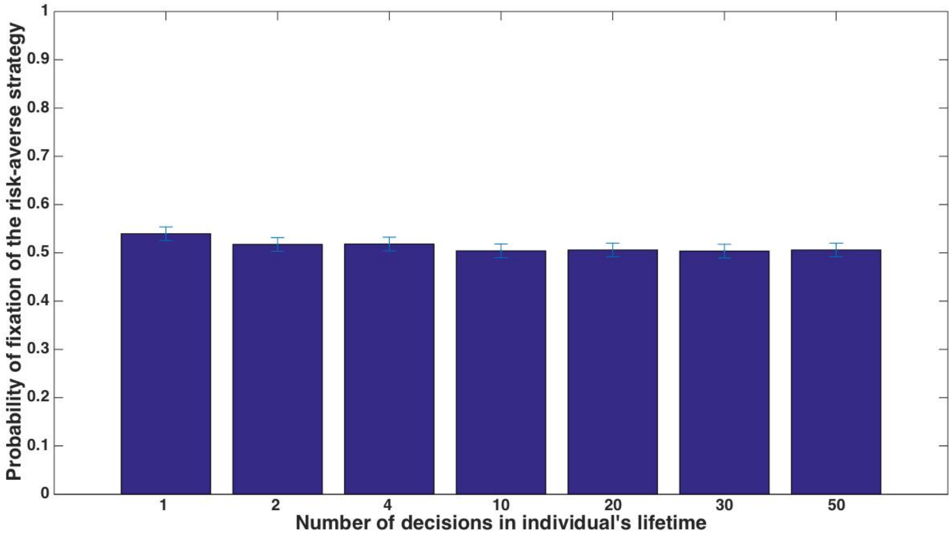

Figure S2: The probability of fixation of the safe strategy under proportional selection, for a payoff scheme of 5-10-15 (risky players receive a payoff of 5 or 15, and a mean of 10; safe players always receive a payoff of 10), with different numbers of decision in an individual's lifetime. The safe strategy has an advantage only when the number of decisions is small: the probability of the safe strategy's fixation decreases from being not significantly different from 0.55, for a single decision during an individual's lifetime, to being only marginally different ($p<0.01$, $p>0.001$) from 0.5 when the number of decisions are 2 and 4, and not significantly different from 0.5 for 10 or more decisions in an individual's lifetime (N=100, 5000 simulations per parameter condition).

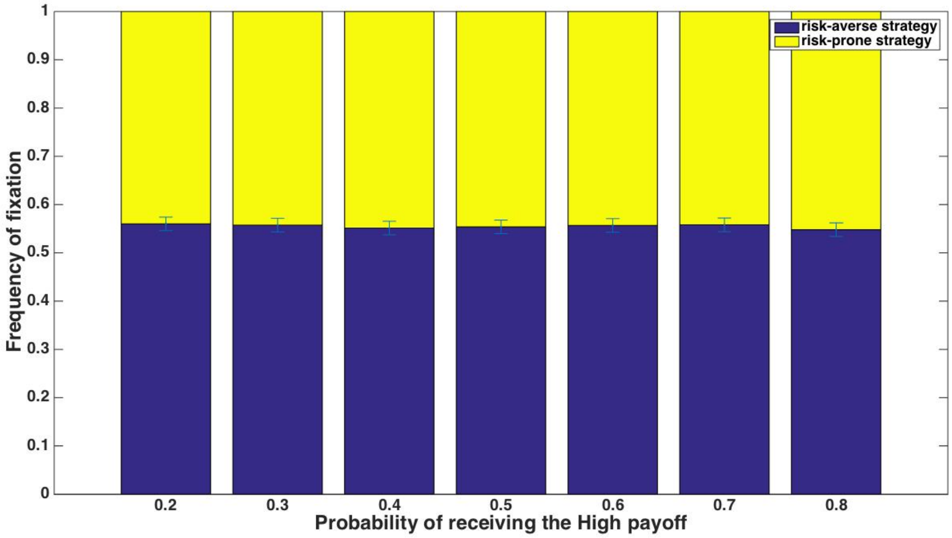

Figure S3: The frequency of fixation of the safe and risky strategies under proportional selection, for different payoff schemes, with a single decision in each individual's lifetime. In all schemes, the mean risky payoff is equal to that of the safe strategy and the variance is the same in all runs. The payoffs of the risky strategy and their probabilities change between conditions. For all conditions, the probability of the safe strategy's fixation is not significantly different from 0.55 (N=100, 5000 simulations per parameter condition).

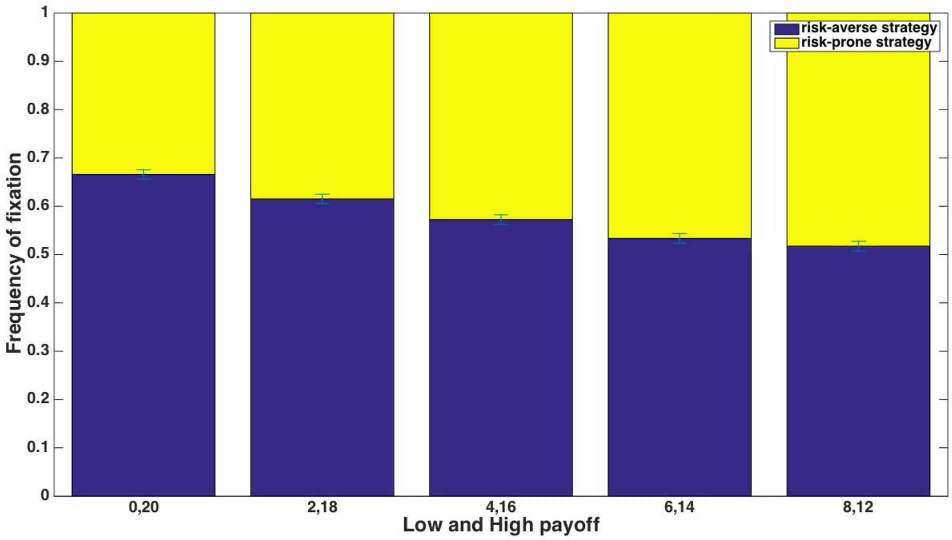

Figure S4: The frequency of fixation of the safe and risky strategies under proportional selection, for different payoff schemes, with a single decision in each individual's lifetime. In all schemes, the mean risky payoff is equal to that of the safe strategy, but the variance in these payoffs is different for each condition. The safe strategy is strongly advantageous when the risky payoff has a high variance, and this advantage decreases as the variance in risky payoffs decreases.

Supplementary section 3: A note on truncation selection

We do not study a simple but important case of truncation selection, because in our framework it leads to a trivial result: the case of truncation at an absolute payoff threshold. Under this scheme, only individuals that accumulated a payoff greater than the threshold reproduce. As opposed to the scenario we studied, in which the threshold value is determined by the distribution of accumulated payoffs in the population, in this case the threshold is constant. This is likely to be a reasonable approximation for the reproductive dynamics in many species, particularly when intra-species competition is low compared to other limiting factors such as inter-species competition, severe climate, or predation. One of the most common thresholds of this sort is body size (e.g. [10–13]).

In our framework the implementation of these reproductive dynamics leads to a trivial result: if the payoff of the risk-averse players is above the set threshold, risk aversion always spreads and fixes in the population, because all individuals that utilize this strategy are selected for reproduction, whereas if the risk-averse players' payoff is below the threshold, this strategy will be lost from the population within a single simulation time step. In the case in which the risk averse payoff is equal to the threshold, the outcome is dependent on a combination of the way the risk averse players are accordingly dealt with (e.g., whether none, all, or half of them are selected for reproduction) and the distribution of the payoffs of the risk-prone strategy. These cases also yield trivial results, and have very limited generality.

These findings, although trivial, lead to a simple but powerful prediction: strong risk preference is expected to evolve when the reproductive threshold is set to a constant value; whether risk aversion or risk proneness would be preferred depends on the value of the payoff distributions. A major caveat of this finding is that this prediction is not readily translatable to predictions about the behavior of most of the species in which a certain body-size is the truncation threshold: body size is typically the product of a very large number of factors, particularly the payoffs of repeated foraging bouts that are on the order of dozens, hundreds, or thousands of foraging events. These conditions are expected to minimize the difference between risk prone and risk averse foraging strategies, as discussed in

the main text, perhaps to an extent that the difference between them is negligible with respect to the reproductive threshold, suggesting that other factors that are not considered in our framework would determine more prominently the behavioral strategy that would be selected.

This scenario also highlights the potential importance of individual state-dependent risk preference, which is not considered in our framework. See *Discussion*.

Supplementary section 4: Realized fitness curves

A parent individual is probabilistically assigned to every individual in a new generation of the simulation from among the individuals of the previous generation. This is done as a function of each individual's accumulated payoff, weighted and normalized according to the reproduction scheme that is implemented (see Methods) and normalized such that the sum of weighted accumulated payoffs of all individuals in the population is equal to 1. Accordingly, each individual's realized fitness depends on the strategies used by the rest of the population and their stochastic payoff during that generation; in the sigmoid-weighted selection scheme, the location of the fitness function's inflection point also depends on the payoff of the single best-performing individual in the population, whose fitness is defined as equal to 1 (see Methods).

Below are a number of examples of concave, convex, and sigmoid fitness functions, as they would have been realized for a range of payoff values following the first generation of a simulation run, when the distribution of strategies of individuals in the population is such that half of the individuals are risk-averse and half are risk-prone, and when the population is large enough such that the distribution of risky payoffs is realized fairly uniformly (i.e. both high and low payoffs for the risky strategy players were (stochastically) well-represented among the payoffs received by players during that generation). Note that the function shows the fitness value of a range of accumulated payoffs, but in the simple risk scenario that is reported in this study only a few values were realized (e.g., in a simulation run in which there is a single event in each individual's lifetime, the payoff received by a risky player can be either the low or the high payoff). Further simulations were conducted to ensure that this discrete distribution does not lead to qualitative artifacts. This simple payoff scheme was preferred for simplicity and increased tractability.

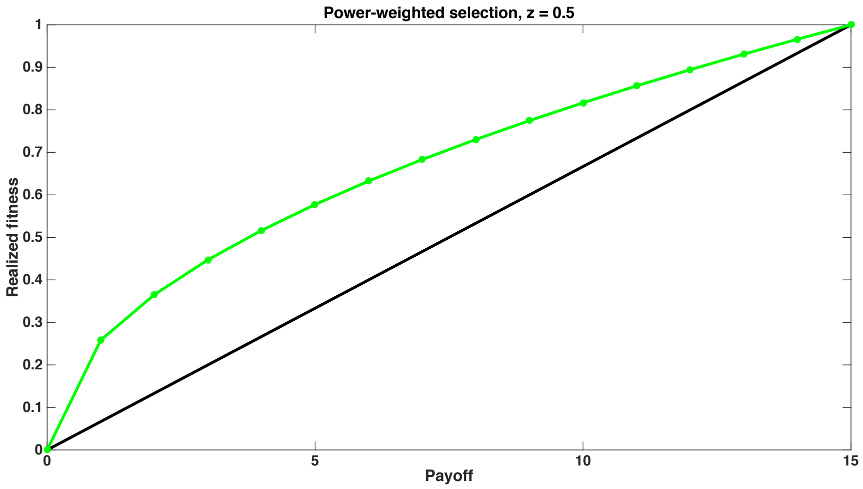

Figure S5: Realized fitness under power-weighted selection, z=0.5.

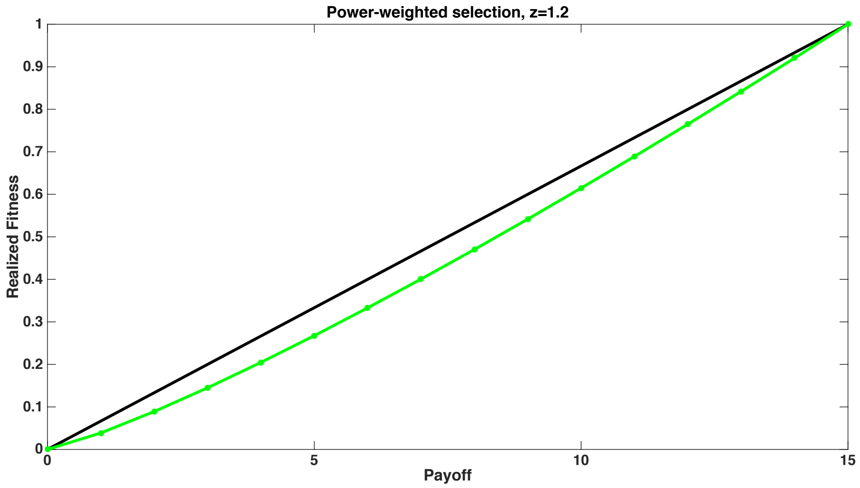

Figure S6: Realized fitness under power-weighted selection, z=1.2.

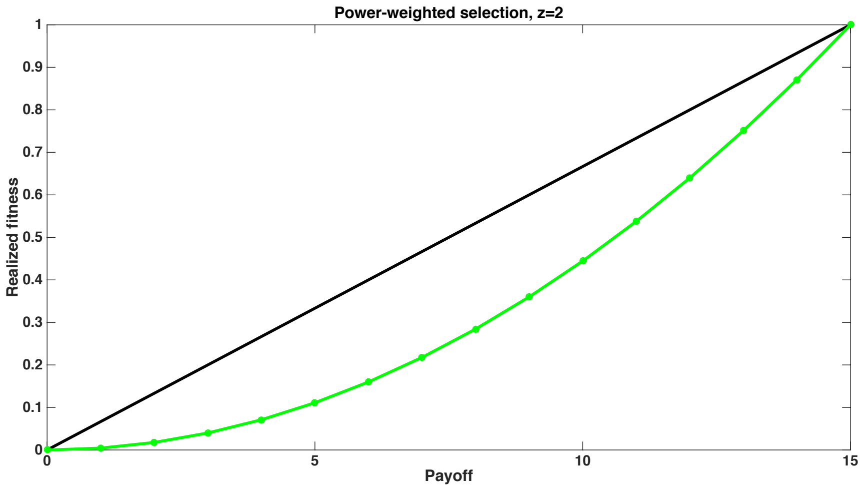

Figure S7: Realized fitness under power-weighted selection, z=2.

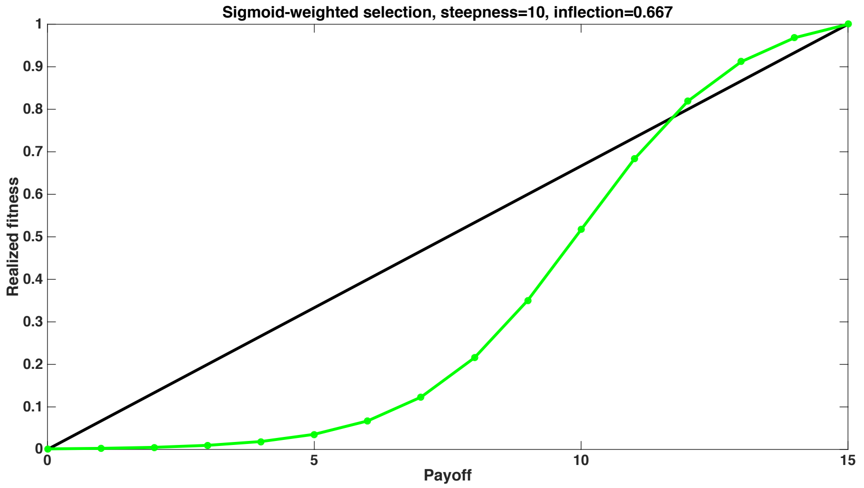

Figure S8: Realized fitness under sigmoid-weighted selection, steepness=10, inflection point = 0.667.

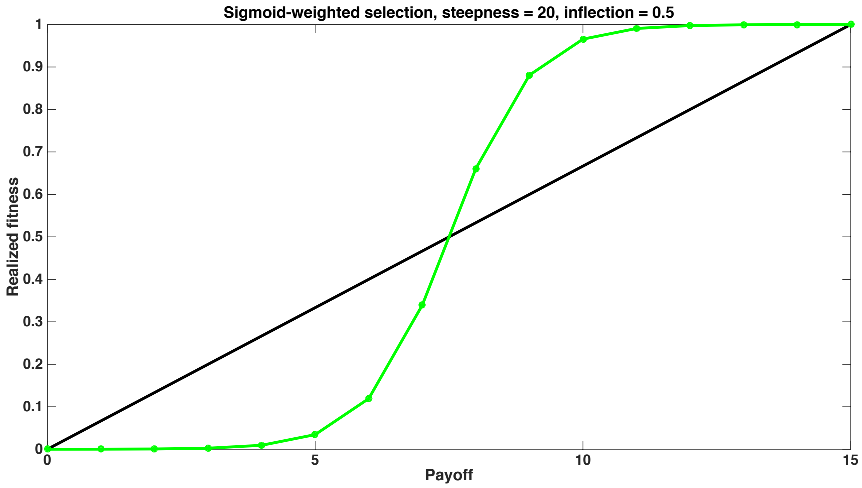

Figure S9: Realized fitness under sigmoid-weighted selection, steepness=20, inflection point = 0.5.

Supplementary section 5: the effect of population finiteness

In each of the scenarios that we studied, some of our findings diverge from the prevalent portrayal of risk taking. Some of these are simply a result of our exploration of reproductive dynamics that are different from those usually considered. Beyond these, a primary factor that leads to divergence from the common dogma is the explicit consideration of a population context. Here we briefly highlight a few of these cases, and compare our results with the prediction of risk preference in the absence of a population context.

1. Proportional selection: we find that a risk averse strategy is preferred, but even when the difference in variance between the two strategies is large, the risk-seeking strategy fixes in a very large minority of cases. This finding is in line with existing theory [8], but is largely under-appreciated, as the discussion frequently focuses on which strategy is preferred, and not on its probability of fixation. In an infinite population a preference for one strategy would not arise at all, as long as the variance is not correlated among individuals [14].
2. Truncation selection: we find that strategy preference is sensitive both to the truncation threshold and to the payoff distribution, including to aspects of it beyond the variance per se. These findings are not unexpected, but are largely under-appreciated, as is evidenced by the common use of truncation selection in computational evolutionary simulations without explicit consideration of the expected effects highlighted by our findings. The truncation scheme that we apply is meaningful only in a population context, as it is dependent on the rank order of the individuals' payoffs. In the absence of a population context, a truncation scheme with a constant threshold may be applied and leads to a potentially different, trivial, outcome, as discussed in supplementary 3.
3. Power-weighted selection (concave/convex fitness functions): we find strong dependence of the outcome on population size (i.e. the disadvantageous strategy may fix in many simulations, dependent on

population size), and even a reversal of the expected preference between the two strategies in scenarios in which the probability of receiving the high or the lower payoff is very low and the population size is small. In the absence of a population context, neither of these phenomena occur.

4. Sigmoid-weighted selection: the shape of the sigmoid in our model depends, as in classic evolutionary studies, on the fitness of the best-performing individual in the population (which is defined to have fitness equal to 1); thus, it is inherently dependent on the population context. We find a qualitative recapitulation of results found in the truncation selection scenario, and most importantly we find that no full generalization can be made regarding whether this scenario would favor risk aversion or risk seeking: for any given sigmoid fitness function, payoff distributions can be found that would favor risk-taking or risk aversion.

Supplementary section 6: A few comments that may help navigate related theory

A review of the literature on the topics of risk taking and bet hedging is beyond the scope of the current study (see, e.g., [14–18]). Here we provide a number of notes that may help understand the links between different approaches in the existing literature and may help to clarify our model's relation to these.

Variance in the fitness-determining outcomes of alternative strategies has been studied in evolutionary and behavioral ecology within two prominent paradigms: risk-sensitivity and bet hedging. Risk sensitivity draws on notions from economics and game theory ([19–21]), corresponds with studies in psychology, neuroanatomy, and cognition ([22–27]), and typically depicts the question of risk sensitivity in the context of behavioral decision-making ([17]), although the preferences and the decision-rules may or may not be genetically-determined. With some important exceptions, much of the risk-taking literature focuses on optimality considerations, studied from the perspective of the individual, and historically was portrayed in many studies in the context of foraging, although most studies aspire to greater generality than this particular context. In line with the focus on the individual organism's perspective, models in this paradigm typically consider time scales that range from minutes to an individual's life span.

The study of bet hedging deals with a similar question – how organisms cope with variance in fitness-related outcomes – from a perspective that focuses on life history and particularly on strategies of reproduction ([14,16,28]). It draws mostly on theory in evolutionary biology, with less obvious links to other disciplines. Accordingly, it is more common in bet hedging than it is in risk-sensitivity that behavior is studied in a population context, with explicit consideration of the fitness associated with alternative strategies and with consideration of evolutionary timescales on the order of generations.

Perhaps the most common depiction of the question in risk-sensitivity frameworks is that of an animal that forages on a limited energy budget and needs to maximize its gains, typically towards a short-term goal such as crossing an accumulated energy threshold in order to survive the night (but not always).

Accordingly, the fitness function assumed is usually either a constant step function (i.e., the threshold for survival is a pre-determined constant) or a concave function with diminishing returns (see [15], table 1, for a partial summary; some such examples are in [17,29]). Frequently, the key to the expected preference stems from Jensen's inequality, supporting risk aversion, and risk preference depends on whether the mean expected payoff of the risk-averse strategy is sufficient to cross the survival threshold (and then it is preferred) or not ([18]). Models that relate risk-sensitivity in the context of foraging to long-term fitness, in the context of reproduction, high rates of background predation, or migration, for example, are few, but some such attempts have been made, e.g. [15,30–32].

Bet hedging can be explained by considering two possible states of the environment and three phenotypes that fare in each of the environments differently. For simplicity, we may refer to the two possible environmental states as dry and wet (following Starrfelt and Kokko, *S&K*, [14]), with inter-generational variability, and the phenotypes as a wet-specialist, a dry-specialist, and a generalist (see also [33]). An evolutionary strategy that does not include bet-hedging would be, in these terms, one in which a lineage specializes to one of the two environments, producing always the dry-specialized or always the wet-specialized phenotype. Bet hedging can be of one of two types: a *conservative* bet-hedging strategy, in which all individuals have the generalist phenotype, and do reasonably well, but not very well, in both environments, and a *diversifying* bet-hedging strategy, in which each individual always has one of the specialized phenotypes, but whose genotype carries the potential for both, and thus each individual's offspring are distributed in their phenotype between the two specialized phenotypes, each with some pre-determined probability (that may be adapted to the probability of each environmental condition's occurrence). This is the type of bet-hedging most frequently studied (see, e.g., [34–36]).

A fundamental principle that underlies many of the bet hedging results is that when there is between-generational variation in the number of offspring, the measure to be optimized is the geometric mean fitness, and not the arithmetic mean ([8,37–39]). Thus, bet hedging frameworks typically clarify early on that variance

minimization would be selected for, and so the arithmetic mean payoff is traded off in favor of decreased variance among the lineage's individuals' payoffs and/or in favor of decreased correlation in the reproductive success among these individuals [14,16]. Notably, though, this relies on approximations that assume that populations are infinite, and it is incorrect to expect the strategy that maximizes the geometric mean to always fix in small populations [8,16,28]. A fundamental reason that decreased variance is selected for is that, in a finite population, each strategy's frequency in the population is a concave function of its payoffs, referred to also as *absolute fitness* ([14,38]). Variance minimization is thus explained by Jensen's inequality.

A dimension of importance in bet hedging modeling is the environmental setting: the environmental state in a given generation may be fully coupled among all individuals (i.e. all experience either dry or wet conditions), may be completely independent for each individual, or may be any intermediate option, in which case the environment can be viewed as spatially heterogeneous, composed of wet and dry patches. The probabilities with which each environmental state occurs – in space and in time – are parameters of the model, and typically the strategies are considered to evolve to adapt accordingly. *S&K* provide a very useful review of the topic, and highlight that the two types of bet hedging (conservative and diversifying) are actually the endpoints of a single spectrum, as are within-generation and between-generation variance, if stochastic within-generation heterogeneity in individuals' payoffs is considered among players of the same strategy.

Most models of bet hedging do not consider a reproductive scenario in which payoffs are translated to fitness via a scheme other than proportional selection, carried out either at the level of the cumulative absolute fitness assigned to each strategy/allele, or at the level of individuals. Alternatively, one might view some models as operating on realized fitness and variance in fitness after the proper reproductive scheme had been applied. Such application is not easily implementable, is rarely – if ever – done, and it is unclear to us whether the underlying assumptions of these models' derivations hold for these cases (such models might be, for example, [14,16,33,38]). In risk-taking, models typically do not explicitly take into account any population-level reproduction dynamics at all.

Our model is portrayed in terms of risk sensitivity, as it aims to highlight the role of variance in payoffs in a broad range of situations that organisms face and in which alternative strategies vary in the variance associated with them; this includes reproduction strategies, as is the focus of many bet hedging models, but also many other behaviors such as dispersal, mate choice, choice of a breeding site, seasonal migration, group affiliation, exploratory behavior, attitude towards novel objects, coping with predation, and foraging. Also, our model is aimed at a broad audience from a number of disciplines, some of which may be more familiar with the terminology of risk sensitivity, and is structured as a comparison between two simple alternative behaviors – risk-prone and risk-averse – to ease its tractability and intuitive understanding.

The setting that we study can also be portrayed in terms of bet hedging. Our risk-averse strategy is analogous to a generalist bet-hedger, or conservative bet hedging, while our risk-seeking strategy with the context in which it is studied can be viewed in one of two ways. In the first, it is viewed as a single-environment specialist, but whose offspring experience completely independent environmental conditions from one another in each choice throughout their lifetime (the probability of experiencing each type of environment is the probability of receiving each of the alternative risky payoffs in our model). The second view of the risk-seeking strategy is as a diversifying bet hedger, whose offspring are split between the two specialized phenotypes with a certain probability, and who all experience the same environment. This interpretation is appropriate only in the case of one event per life time, as it requires that the offspring of a certain phenotype remain coupled in the environment that they experience throughout their lifetime, and in our model the correlation between these individuals will not be maintained over more than one event. Importantly, both of the strategies in our study experience the same mean payoff and differ in variance; in bet hedging studies, strategies typically differ from one another in the arithmetic mean payoff that they experience.

The findings that we highlight in this study – in particular the prominent role that reproduction dynamics and other aspects of the population context play in determining evolutionary outcomes – are very general in their applicability and

could be explored and demonstrated even in the simple setting that we chose: it was sufficient in order to show that reproduction strategies which are commonly discussed and which are more realistic, for many species, than proportional selection, yet were never considered in the context of risk sensitivity or bet hedging as far as we know, have a profound effect on evolutionary outcomes. If such studies have been carried out, they seem to have had a limited effect on the perception of these topics in a number of sub-disciplines of evolutionary biology. The finding that these deviations from the existing paradigm have far-reaching implications for selection on risk sensitivity will probably not come as a surprise to theoreticians that study risk sensitivity or bet hedging, yet we believe that their explicit study in a simple setting in which results can be intuitively explained may aid in the appreciation of their importance among the broader scientific community.

Supplementary section 7: The relation between risk-related behaviors that occur in key points of life and risk-related behaviors that recur frequently

Our study focuses on the effect of risk-related behavior in key points in an individual's life history in which the behavior in one or few events may have large long-term fitness consequences. We find – not surprisingly – that a context in which payoff is summed across multiple events leads to a minimization of the difference between the overall payoff of risk-prone and risk-averse strategies, an effect that can be explained by the law of large numbers. As opposed to such contexts, much of the classical treatment of risk taking has focused on frequently-recurring foraging behavior (e.g. [17,31,40–45]). Linking the outcomes of foraging to long-term fitness, as measured by – for example – lifetime reproductive success, is hard (e.g., [46]), and is beyond the scope of the current study. The following points may help to map out the relation between different types of risk-related behavior and their predicted effect on selection on risk preference.

- To explain risk-sensitivity in foraging contexts, researchers have posited that the animals are attempting to optimize different goals, that may be linked to long-term fitness in various ways: an individual may be under a constant energy-budget constraint, for example, and may run the risk of not surviving to the next day if it does not gather enough food to exceed a certain energy threshold (e.g., [44,47,48]). This scenario is very realistic for some species, such as many passerine birds.
- Apparent risk-sensitivity in foraging has been proposed to stem in some cases from differences in individual experience, leading individuals to estimate the distribution of payoffs from alternative strategies differently from one another, and perhaps differently from the true distributions (e.g. [49–54]). This scenario suggests that, in some cases, different individuals would learn to prefer different strategies – and seem to have opposite risk-taking preferences – solely as a consequence of their different experiences.
- Risk preference in foraging tasks (or tasks similar to them in their being characterized by many events) can perhaps be explained by the cognitive

underpinnings of the choice of behavior (e.g. [27,49,55]), for example the application of domain-general cognitive mechanisms to a foraging task may lead to risk-aversion that has no advantage in the studied context, but does have an advantage in other contexts that influenced the cognitive mechanism's evolution. These include other foraging contexts, perhaps different from the one that is studied.

- It is quite possible that the complex reality of animals' day-to-day life creates interdependency between different tasks that we tend to perceive as separate. Thus, for example, it may be that an animal is not at risk of not surviving the night because of lack of energy, but is still forced to optimize its short-term accumulation of resources because the risk of predation is different depending on the body-size attained within a short period of time. Such a constraint can lead to strong risk-related preferences (and could lead to either risk-aversion or risk-proneness, depending on the specific ecology).
- A situation in which foraging payoff may impact the outcomes of near-future success or survival – for example via short-term gain of body-weight or available energy – may bring about strong selection on risk preference. Thus, for example, if competition for resources is severe, the likelihood of succeeding in one foraging bout may strongly depend on success in the one that precedes it. Similarly, success in an aggressive interaction between males may be dependent on success in the previous interaction. A setting like this is similar to the dynamics of compounding interests in economics: small differences in payoffs at an early stage may lead to disproportional effects on an individual's long-term fitness.

Supplementary references

1. Shuster, S. M. in *Evolutionary Behavioral Ecology* (eds. Fox, C. & Westneat, D. F.) 434–450 (Cambridge University Press).
2. Gueijman, A., Ayali, A., Ram, Y. & Hadany, L. Dispersing away from bad genotypes: the evolution of Fitness-Associated Dispersal (FAD) in homogeneous environments. *BMC Evol. Biol.* **13,** 125 (2013).
3. Hadany, L., Eshel, I. & Motro, U. No place like home: competition, dispersal and complex adaptation. *J. Evol. Biol.* **17,** 1328–1336 (2004).
4. Hamilton, W. D. & May, R. M. Dispersal in stable habitats. *Nature* **269,** 578–581 (1977).
5. Roff, D. A. The evolution of flightlessness in insects. *Ecol. Monogr.* **60,** 389–421 (1990).
6. Frank, S. A. Dispersal polymorphisms in subdivided populations. *J. Theor. Biol.* **122,** 303–309 (1986).
7. Johnson, M. L. & Gaines, M. S. Evolution of dispersal: theoretical models and empirical tests using birds and mammals. *Annu. Rev. Ecol. Syst.* **21,** 449–480 (1990).
8. Gillespie, J. H. Natural selection for within-generation variance in offspring number. *Genetics* **76,** 601–606 (1974).
9. Ewens, W. J. *Mathematical Population Genetics 1: Theoretical Introduction*. **27,** (Springer Science & Business Media, 2012).
10. Naulleau, G. & Bonnet, X. Body condition threshold for breeding in a viviparous snake. *Oecologia* **107,** 301–306 (1996).
11. Weimerskirch, H. Reproductive effort in long-lived birds: age-specific patterns of condition, reproduction and survival in the wandering albatross. *Oikos* **64,** 464 (1992).
12. Bagamian, K. H., Heins, D. C. & Baker, J. A. Body condition and reproductive capacity of three-spined stickleback infected with the cestode *Schistocephalus solidus*. *J. Fish Biol.* **64,** 1568–1576 (2004).
13. Chastel, O., Weimerskirch, H. & Jouventin, P. Influence of body condition on

reproductive decision and reproductive success in the blue petrel. *Auk* **112,** 964–972 (1995).

14. Starrfelt, J. & Kokko, H. Bet-hedging—a triple trade-off between means, variances and correlations. *Biol. Rev.* **87,** 742–755 (2012).
15. Bednekoff, P. A. Risk-sensitive foraging, fitness, and life histories: where does reproduction fit into the big picture? *Am. Zool.* **36,** 471–483 (1996).
16. Frank, S. A. & Slatkin, M. Evolution in a variable environment. *Am. Nat.* 244–260 (1990).
17. McNamara, J. M. & Houston, A. I. Risk-sensitive foraging: a review of the theory. *Bull. Math. Biol.* **54,** 355–378 (1992).
18. Smallwood, P. D. An introduction to risk sensitivity: The use of Jensen's inequality to clarify evolutionary arguments of adaptation and constraint. *Am. Zool.* **36,** 392–401 (1996).
19. Von Neumann, J. & Morgenstern, O. Theory of games and economic behavior, 2nd rev. (1947).
20. Kahneman, D. & Tversky, A. Prospect theory: An analysis of decision under risk. *Econom. J. Econom. Soc.* 263–291 (1979).
21. Markowitz, H. M. *Portfolio Selection: Efficient Diversification of Investments*. (Yale University Press, 1959).
22. Symmonds, M., Bossaerts, P. & Dolan, R. J. A behavioral and neural evaluation of prospective decision-making under risk. *J. Neurosci.* **30,** 14380–14389 (2010).
23. Christopoulos, G. I., Tobler, P. N., Bossaerts, P., Dolan, R. J. & Schultz, W. Neural correlates of value, risk, and risk aversion contributing to decision making under risk. *J. Neurosci.* **29,** 12574–12583 (2009).
24. Trepel, C., Fox, C. R. & Poldrack, R. A. Prospect theory on the brain? Toward a cognitive neuroscience of decision under risk. *Cogn. brain Res.* **23,** 34–50 (2005).
25. Hintze, A., Olson, R. S., Adami, C. & Hertwig, R. Risk sensitivity as an evolutionary adaptation. *Sci. Rep.* **5,** 8242 (2014).
26. Hertwig, R. & Erev, I. The description–experience gap in risky choice. *Trends*

Cogn. Sci. **13,** 517–523 (2009).

27. Hertwig, R., Barron, G., Weber, E. U. & Erev, I. Decisions from experience and the effect of rare events in risky choice. *Psychol. Sci.* **15,** 534–539 (2004).
28. King, O. D. & Masel, J. The evolution of bet-hedging adaptations to rare scenarios. *Theor. Popul. Biol.* **72,** 560–575 (2007).
29. Cartar, R. V & Dill, L. M. Why are bumble bees risk-sensitive foragers? *Behav. Ecol. Sociobiol.* **26,** 121–127 (1990).
30. Gillespie, R. G. & Caraco, T. Risk-Sensitive Foraging Strategies of Two Spider Populations. *Ecology* **68,** 887–899 (1987).
31. McNamara, J. M., Merad, S. & Houston, A. I. A model of risk-sensitive foraging for a reproducing animal. *Anim. Behav.* **41,** 787–792 (1991).
32. Hurly, A. T. The twin threshold model: risk-intermediate foraging by rufous hummingbirds, Selasphorus rufus. *Anim. Behav.* **66,** 751–761 (2003).
33. Philippi, T. & Seger, J. Hedging one's evolutionary bets, revisited. *Trends Ecol. Evol.* **4,** 41–44 (1989).
34. Garcia-Gonzalez, F., Yasui, Y. & Evans, J. P. Mating portfolios: bet-hedging, sexual selection and female multiple mating. *Proc. R. Soc. London B Biol. Sci.* **282,** 20141525 (2015).
35. Simovich, M. A. & Hathaway, S. A. Diversified bet-hedging as a reproductive strategy of some ephemeral pool anostracans (Branchiopoda). *J. Crustac. Biol.* **17,** 38–44 (1997).
36. Watson, P. J. Multiple paternity as genetic bet-hedging in female sierra dome spiders, Linyphia litigiosa (Linyphiidae). *Anim. Behav.* **41,** 343–360 (1991).
37. Gillespie, J. H. Natural selection with varying selection coefficients–a haploid model. *Genet. Res.* **21,** 115–120 (1973).
38. Gillespie, J. H. Natural selection for variances in offspring numbers: a new evolutionary principle. *Am. Nat.* **111,** 1010–1014 (1977).
39. Cohen, D. Optimizing reproduction in a randomly varying environment. *J. Theor. Biol.* **12,** 119–129 (1966).
40. Caraco, T. Risk-sensitivity and foraging groups. *Ecology* **62,** 527–531 (1981).
41. Caraco, T. Energy budgets, risk and foraging preferences in dark-eyed juncos

(Junco hyemalis). *Behav. Ecol. Sociobiol.* **8,** 213–217 (1981).

42. Caraco, T., Martindale, S. & Whittam, T. S. An empirical demonstration of risk-sensitive foraging preferences. *Anim. Behav.* **28,** 820–830 (1980).
43. Caraco, T. White-crowned sparrows (Zonotrichia leucophrys): foraging preferences in a risky environment. *Behav. Ecol. Sociobiol.* **12,** 63–69 (1983).
44. Barnard, C. J., Brown, C. A. J., Houston, A. I. & McNamara, J. M. Rick-sensitive foraging in common shrews: an interruption model and the effects of mean and variance in reward rate. *Behav. Ecol. Sociobiol.* **18,** 139–146 (1985).
45. Stephens, D. W. & Krebs, J. R. *Foraging theory*. (Princeton University Press, 1986).
46. Houston, A., Clark, C., McNamara, J. & Mangel, M. Dynamic models in behavioural and evolutionary ecology. *Nature* **332,** 29–34 (1988).
47. McNamara, J. M. Risk-prone behaviour under rules which have evolved in a changing environment. *Am. Zool.* **36,** 484–495 (1996).
48. Stephens, D. W. & Charnov, E. L. Optimal foraging: some simple stochastic models. *Behav. Ecol. Sociobiol.* **10,** 251–263 (1982).
49. Kacelnik, A. & Bateson, M. Risky theories—the effects of variance on foraging decisions. *Am. Zool.* **36,** 402–434 (1996).
50. Mallpress, D. E. W., Fawcett, T. W., Houston, A. I. & McNamara, J. M. Risk attitudes in a changing environment: An evolutionary model of the fourfold pattern of risk preferences. *Psychol. Rev.* **122,** 364 (2015).
51. Dingemanse, N. J. *et al.* Individual experience and evolutionary history of predation affect expression of heritable variation in fish personality and morphology. *Proc. R. Soc. London B Biol. Sci.* rspb-2008 (2009).
52. Ilan, T., Katsnelson, E., Motro, U., Feldman, M. W. & Lotem, A. The role of beginner's luck in learning to prefer risky patches by socially foraging house sparrows. *Behav. Ecol.* art079 (2013).
53. Katsnelson, E., Motro, U., Feldman, M. W. & Lotem, A. Early experience affects producer–scrounger foraging tendencies in the house sparrow. *Anim. Behav.* **75,** 1465–1472 (2008).
54. Keynan, O. Effect of group size and composition on individual behavior, group

dynamics and population regulation in the Arabian babbler (Turdoides squamiceps). (2015).

55. Gigerenzer, G., Hertwig, R. & Pachur, T. *Heuristics: The foundations of adaptive behavior*. (Oxford University Press, Inc., 2011).
